# Butuanimides, Fatty Acid Synthesis-Inhibiting Antibiotics from Symbiotic Bacteria

**DOI:** 10.1021/acschembio.6c00130

**Published:** 2026-04-28

**Authors:** Bailey W. Miller, Albebson L. Lim, Jeannie Bailey, Mark Jeremiah B. Cleofas, Noel Lacerna, Marvin A. Altamia, Jared T. Seale, Jose Miguel D. Robes, Hiroaki Naka, Colin Manoil, Margo G. Haygood, Eric W. Schmidt, Gisela P. Concepcion

**Affiliations:** † Department of Medicinal Chemistry, 7060 University of Utah, Salt Lake City, Utah 84112, United States; ‡ Department of Genome Sciences, 7284 University of Washington, Seattle, Washington 98195, United States; § The Marine Science Institute, University of the Philippines, Diliman, Quezon City 1101, Philippines

## Abstract

With the ongoing antibiotic drug resistance crisis, new molecules with new mechanisms of action are essential. Here, we characterized quorum sensing-regulated butuanimides from symbiotic γ-proteobacteria, *Teredinibacter* sp. 2052S, which kill Gram-positive bacterial and human cells with micromolar and submicromolar potencies, respectively. Butuanimides share a peptide-imide moiety with andrimid-class antibiotics that target bacterial acetyl-CoA carboxylase (ACC), the rate-limiting step in fatty acid biosynthesis. Similarly, site-directed mutagenesis in *Acinetobacter baylyi* identified the ACC carboxyl transferase (CT) subunit as responsible for butuanimide antibacterial activity. The andrimid-like peptide-imide moiety is attached to a longer, halogenated polyene chain that initiates with an unusual starter unit likely derived from phenylalanine. The resulting epoxyquinone is unstable in solution over a period of hours to days, enabling redox control of antibiotic action. Comparison of the hybrid polyketide synthase-nonribosomal peptide synthetase (PKS-NRPS) biosynthetic gene clusters of butuanimides and andrimid suggests the repurposing of a key phenylalanine-derived motif. The butuanimide structures link the thailandamide- and andrimid-class ACC inhibitors, which should aid ongoing efforts in the development of ACC inhibitors to treat multidrug-resistant infections.

## Graphical abstract

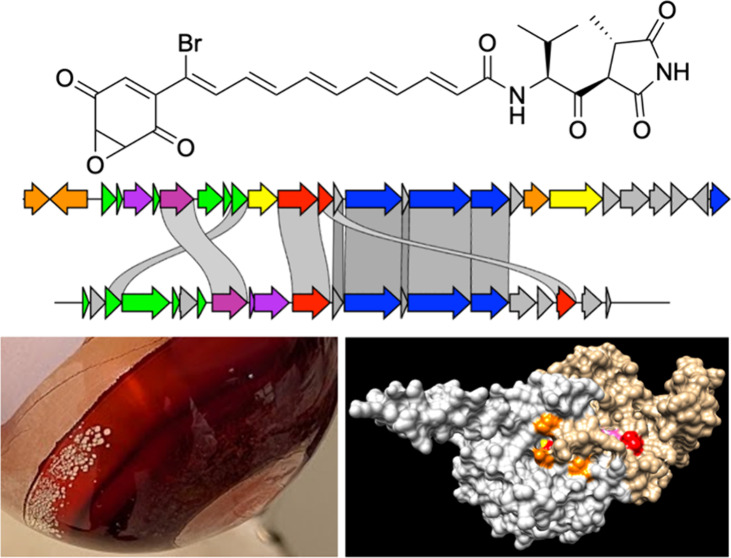

## 1 Introduction

Fatty acid biosynthesis inhibitors include approved antibiotics and pharmaceutical leads for the multidrug resistance crisis.^1^ Among these, several compounds target the acetyl-CoA carboxylase (ACC) complex, which catalyzes the conversion of acetyl-CoA to malonyl-CoA, the rate-limiting step in fatty acid biosynthesis.^2–5^ ACC inhibitors have found use as therapeutic leads for several conditions,^6,7^ including several natural products that target the bacterial ACC (Figure 1). Andrimid (**1**) and moiramide B (**2**) are structurally related bacterial metabolites that act selectively on bacterial ACC, while the hydroxylated analog moiramide C (**3**) is inactive. Thailandamides A and B (**4**, **5**), as well as equisetin (**6**) and pyrrolocin C (**7**), target bacterial ACC but are also toxic to human cells.^5,8^ Compounds **1**, **2**, **4**, and **5** bind to the AccA and AccD heterodimer, blocking the carboxyl transferase (CT) reaction,^9^ while **6** and **7** block biotin carboxylation by AccB/AccC. The binding sites of **1**, **2**, **4**, and **5** on AccA/D were mapped by X-ray crystallography or mutagenesis, providing a precise understanding of molecular mechanisms.^4,5,9,10^

**Figure 1:**
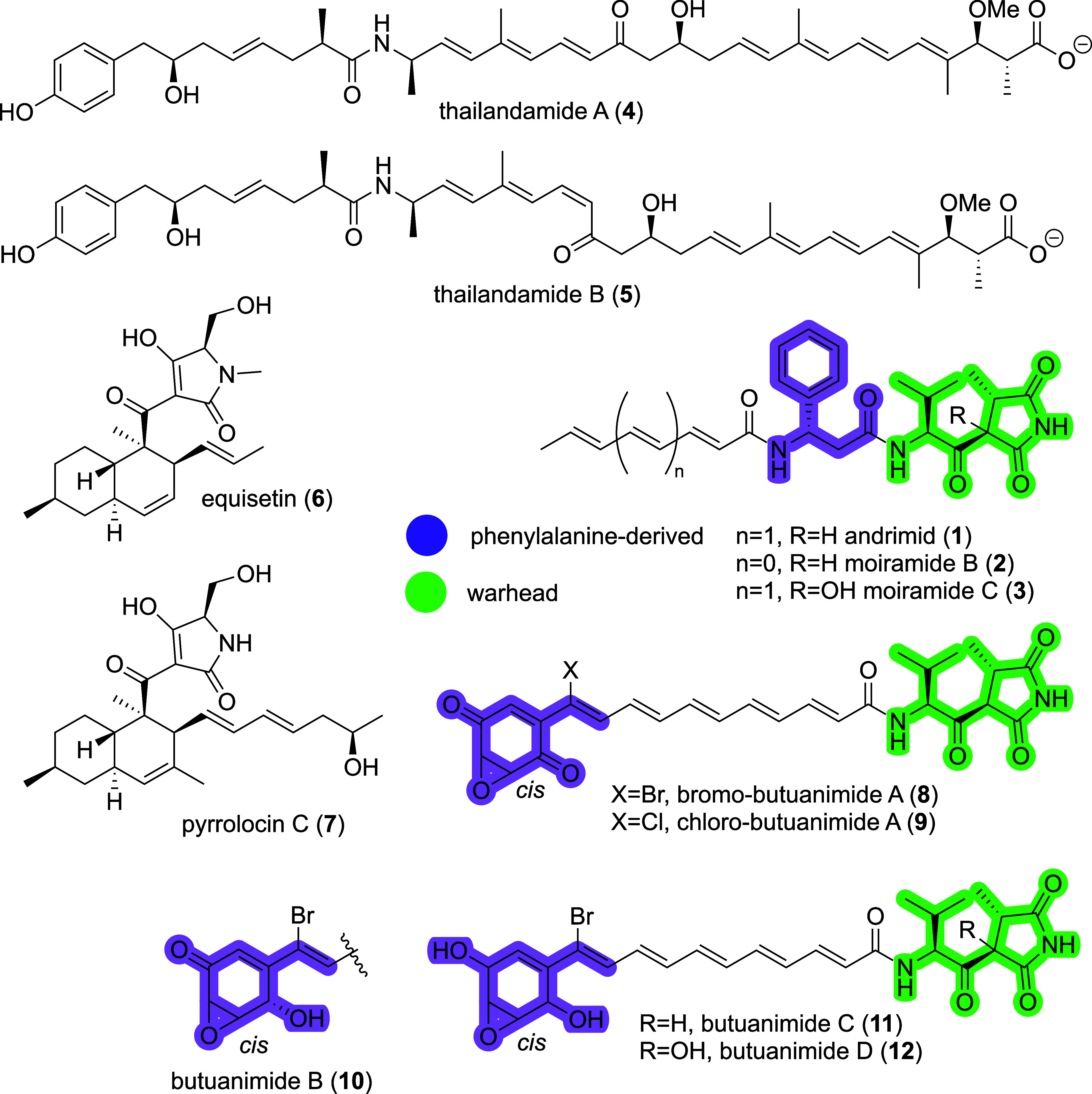
Natural product acetyl-CoA carboxylase (ACC) inhibitors. The activities of compounds **1–7** targeting ACC were previously described, while in this work we define the anti-ACC functions of **8** and **9**. Compounds shown are antibacterial, with the exceptions of **11** and **12,** which were inactive in initial studies.

The apparent selectivity of **1** and **2** for bacterial ACC spurred significant innovation in creating analogs through synthetic and biosynthetic means.^10–16^ As a result, several compounds with greater potency, selectivity, and/or cell penetrance in comparison to the natural products have been described. However, since the publication of moiramide B (**2**) in 1994, there have been no further reports of natural products in this class, limiting the scope of structure–activity studies. To the best of our knowledge, no analog of **1** or **2** has yet entered clinical development.

Here, we report a group of ACC inhibitory natural products, the butuanimides (**8–12**), which expand the chemical space available for further innovation in fatty acid biosynthesis inhibitor design (Figure 1). Butuanimides were isolated from the animal symbiotic microbiome, which is increasingly recognized as a source of antibiotics with novel scaffolds. In 2008, we initiated an NIH-sponsored program, the Philippine Mollusk Symbiont International Cooperative Biodiversity Group (PMS-ICBG), as an early step to capturing this relatively untapped biological and chemical diversity. This decade-long collaboration led by laboratories at the University of the Philippines and in the United States cultivated true animal symbionts, yielding potent natural products.

The PMS-ICBG team isolated butuanimide-producing strain *Teredinibacter* sp. 2052S.S.stab0a.01 (hereafter, “2052S”) from the gills of the marine shipworm *Bactronophorus* cf. *thoracites* collected near Butuan in northeastern Mindanao, Philippines.^17^ *Teredinibacter* symbionts live mostly within the animal gill bacteriocyte cells, where they produce abundant carbohydrate-active enzymes (CAZymes). Remarkably, the enzymes are transported from the gill to a relatively axenic cecum, enabling host animals to consume wood.^18–20^ Unlike most intracellular symbionts, shipworm symbionts such as *Teredinibacter* can be cultured, enabling direct exploration of symbiotic chemical biology.^18^ Indeed, shipworm symbiont genomes encode a remarkable diversity and density of biosynthetic gene clusters (BGCs), comparable to some of the most prolific *Streptomyces* strains,^21^ and several potent antibiotics and other metabolites have already been reported from *Teredinibacter* spp.^22–27^

The butuanimides (**8–12**) from 2052S contain the same andrimid/moiramide B warhead that binds to AccA/AccD. However, the remaining elements have been reshuffled, providing a group of distinct molecules. Among these, butuanimides lack the extender β-phenylalanine moiety of **1**-**3**, instead containing a biochemically related phenylalanine-derived epoxyquinone starter unit that enables redox control of antibiotic activity. Here, we disclose the butuanimide biosynthetic gene cluster (*btm*). The biosynthetic differences between *btm* and the previously reported *adm* cluster illustrate the evolutionary origins of these pathways. They share elements including nonribosomal peptide synthetase (NRPS) and polyketide synthase (PKS) motifs. However, the phenylalanine ammonia lyase (PAL)-encoding gene^28^ has been repurposed for starter unit instead of extender unit synthesis. Butuanimides exhibited modest antibiotic activity, which was traced to specific sites in the AccA/AccD proteins using a mutagenesis strategy. Cytotoxic activity to human cells was slightly stronger than antibacterial activity for all active compounds, particularly the chlorinated analog. These QS-regulated metabolites illustrate how symbiotic bacteria recombine biosynthetic machinery to create new scaffolds, expanding opportunities for ACC inhibitor design.

## 2 Results and Discussion

### 2.1 QS-Linked Compound Production

In 2015, using assay guided fractionation, the PMS-ICBG team identified strong antibacterial activity in the bright red fractions of 2052S extracts. Two pigmented compounds, **11** and **12**, were sufficiently stable, enabling us to elucidate their planar structures. Unfortunately, we could not detect reproducible antibacterial activity in either these compounds or in other fractions, leaving the active metabolites unknown. Recognizing that the active molecules were likely rapidly degrading, the results were not published, and instead the project was shelved for several years.

Subsequently, Puri’s group deleted the 2052S homoserine lactone synthase (*tbaI*), resulting in a loss of red pigmentation compared to the wild-type strain and demonstrating QS control of metabolite production.^17^ With this tool in hand, we compared the HPLC-DAD chromatograms of the wild-type and knockout strains. We observed a striking difference, in which the red color was nearly absent and with several peaks displaying absorption between 300 and 500 nm being severely downregulated in the Δ*tbaI* strain (Figure 2A,B). To trap and isolate unstable compounds that were suspected to degrade during fermentation, sterile Diaion HP-20 resin was added to the culture broth during growth, then collected and eluted under gentle conditions. These extracts were highly enriched in several additional compounds (**8** **–** **10**) with longer wavelength absorbance (300–575 nm) (Figure 2C,D). The compounds could be purified to homogeneity, but they were unstable in pure form; instability seemed to worsen when the pure compounds were more concentrated in solution, underwent freeze–thaw cycles, or were dried and redissolved in new solvent. Compounds **8–10** were active in antibiotic assays (Table 1), and therefore their structures were elucidated.

**Figure 2:**
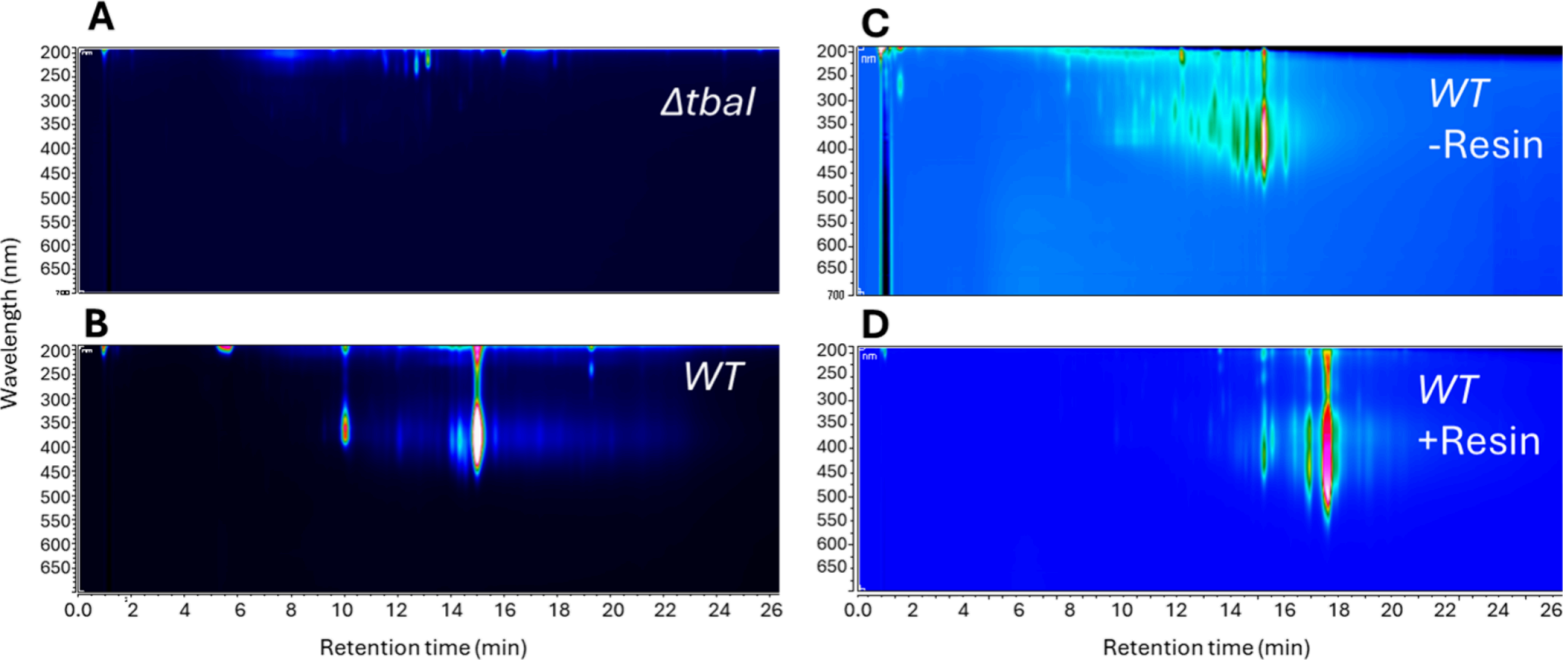
Isolation of butuanimides under different culture conditions. HPLC-diode array detector profiling of extracts. (A) Extract of supernatant from 2052S Δ*tbaI*. (B) Extract of supernatant from WT 2052S. (C) Extract of supernatant from WT grown without resin present during fermentation. (D) Extract of supernatant from WT grown in the presence of hydrophobic resin.

**Table 1:** Bioactivity of Butuanimides^a^

	**MIC**_**90**_ **(μg/mL)**					**IC**_**50**_ **(μg/mL)**
**compound**	AB	EC	SA	KP	EF	HEK293
**8**	>32	>32	8	>32	16	2.8, 4.5
**9**	>32	>32	4	>32	8	0.34, 0.91
10	>32	>32	4	>32	16	4.1, 5.2

a AB: *Acinetobacter baumannii*; EC: *Escherichia coli* C600; SA: *Staphylococcus aureus*; KP: *Klebsiella pneumoniae*; EF: *Enterococcus faecium*; and HEK293: human embryonic kidney 293 cells.

### 2.2 Structure Elucidation of Butuanimides

High-resolution electrospray ionization mass spectrometry (HRESIMS) of **8** indicated a molecular formula of C_27_H_27_N_2_O_7_Br, based on a protonated ion at *m*/*z* 571.1059 [M + H]^+^ (calcd *m*/*z* 571.1080) and an isotopic pattern consistent with bromine incorporation (Figure S1). The ^1^H NMR spectrum revealed features of a polyene structure, including signals integrating to 10 protons between δ 6.3–7.5. Additional signals included 3 doublet methyls, a putative α-proton (δ 4.63), an amide proton (δ 8.38), a potential imide proton (δ 11.38), and several resonances between δ 2.8 and 4.2 ppm. The ^13^C NMR spectrum indicated a ketone (δ 203.8), five additional carbonyls (δ 165.5–191.5), 12 olefinic carbons (δ 117.6–142.8), three methyl (δ 14.8–19.5) and four additional signals. The final carbon resonance was found to overlap with residual DMSO based on HSQC and HMBC correlations.

COSY and TOCSY experiments identified four distinct spin systems in **8**. The first included two methyl doublets (δ 0.85, 0.91), a methine (δ 2.34), an α-proton (δ 4.63), and an amide proton (δ 8.38). COSY and HMBC correlations supported a valine substructure with a ketone carbonyl at δ 203.8 and additional HMBC correlations to a carbonyl at δ 165.5.

The second spin system included a methyl doublet (δ 1.14) and two methine protons (δ 2.89 and 4.02), each showing HMBC correlations to the valine ketone (δ 203.8) and two additional carbonyls (δ 180.2, 174.0). The imide proton (δ 11.38) correlated with both methine carbons (δ 57.8, 39.4) and the carbonyls, supporting a methyl-succinimide moiety adjacent to valine. Comparison of reported ^13^C and ^1^H chemical shifts between compounds **8** and **1** confirmed near-identical values, indicating conserved relative configuration (Table S1).

The third spin system included nine protons between δ 6.35 and 7.51 ppm, consistent with a polyene chain. Six well-resolved signals were assigned via HSQC, HMBC, and NOESY. The most shielded proton (δ 6.35) showed COSY correlation to δ 7.15, and both had HMBC correlations to the carbonyl at δ 165.5, placing this end of the polyene adjacent to the valine N-terminus. A strong NOESY correlation between δ 6.35 and the valine NH further supported the assignment. At the opposite end, the most deshielded proton (δ 7.51) showed a COSY correlation to δ 6.72, and both showed HMBC correlations to a nonprotonated carbon at δ 117.6.

The final TOCSY spin system included one olefinic proton (δ 6.67) and two deshielded methine protons (δ 4.01, 4.15), assigned to carbons δ 54.7 and 54.6 via HSQC. HRMS data indicated that the remaining fragment had a formula of C_7_H_3_O_3_Br and contained five degrees of unsaturation. HMBC correlations from δ 4.15 and δ 6.67 to δ 190.8, and from δ 4.01 to δ 191.4, indicated two ketones. The δ 6.67 proton also correlated with δ 142.8 and δ 117.6, suggesting proximity to the polyene terminus. These data supported an epoxyquinone moiety. The complete structure, including key HMBC and NOESY correlations for connectivity, is shown in Figure 3.

**Figure 3:**
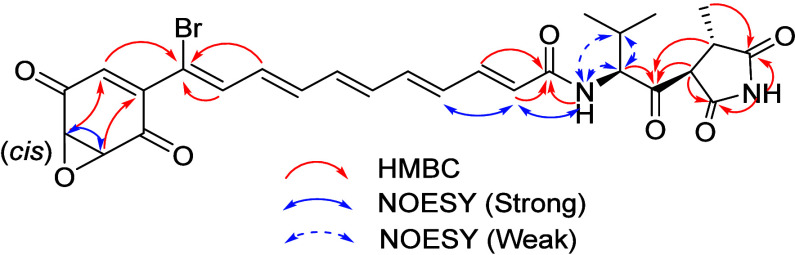
Key 2D NMR correlations used to determine planar structure of bromo-butuanimide A (**8**).

Compound **9** was assigned the formula of C_27_H_27_N_2_O_7_Cl, based on HRESIMS (*m*/*z* 527.1656 [M + H]^+^, calcd for C_27_H_28_N_2_O_7_Cl ^+^, *m*/*z* 527.1585) and an isotopic distribution consistent with chlorine (Figure S9). The ^13^C NMR spectrum for the pure compound **9** was relatively weak, but a combination of a high-quality ^13^C spectrum of a concentrated mixture of **8** and **9** (Figure S12), as well as phase-sensitive HSQC and HMBC data for the pure compounds, enabled assignment of all carbon chemical shifts and clearly differentiated the ^13^C signals for each congener. The ^1^H and ^13^C data matched that of **8** except for minor shifts at positions 21–25, centered around the halogenation site. A shift in the ^13^C signal at position 23 from δ 117.6 to 124.7 is consistent with a substitution of bromine with chlorine.

Compound **10** was brominated and included two additional protons relative to compound **9,** as determined by HRESIMS (*m*/*z* 573.1241 [M + H]^+^, calcd for C_27_H_30_N_2_O_7_Br^+^, *m*/*z* 573.1236) (Figure S18). A ^13^C NMR spectrum was not obtained due to limited material and compound instability, but HMBC and HSQC data was sufficient to assign carbon chemical shifts due to the close structural similarity between compounds **8** and **10**. NMR data for the methyl succinimide and valine moieties were nearly identical to those of **8**, indicating that the site of reduction is the six membered ring. A new resonance at δ 4.93 and shifts of the epoxide protons to δ 3.53 and 3.85 indicated the presence of an epoxyquinol. HMBC correlations from δ 5.82 to the carbinol and one epoxide carbon, δ 4.93 to the olefinic carbon, and the epoxide protons to the carbonyl and carbinol carbons confirmed this structure. The carbinol proton at δ 4.93 appeared as a broadened singlet, indicating an extremely small coupling constant with the adjacent epoxide proton. This supported a *trans* relative configuration between the epoxide and alcohol.^29,30^

Compound **11** had two additional protons compared to **10**, with HRESIMS indicating a formula of C_27_H_31_N_2_O_7_Br based on a protonated ion at *m*/*z* 575.1367 [M + H]^+^ (calcd *m*/*z* 575.1393) and bromine incorporation (Figure S26). The final spin system included five resonances: the epoxide protons and the olefinic proton found compounds **8** and **9,** plus two additional signals at δ 4.35 and δ 4.74. These indicated reduction of both carbonyls to an epoxyhydroquinone, which was supported by COSY and HMBC correlations.

Compound **12** was determined to share the same core structure as compound **11**, including the epoxyhydroquinone headgroup, with one additional oxygen. HRESIMS data supported a molecular formula of C_27_H_31_N_2_O_8_Br (*m*/*z* 591.1339 [M + H]+, calcd *m*/*z* 591.1337). Compared to compound **11**, the signal at δ 4.02 was absent, and the succinimide methyl group exhibited a new correlation to a carbon at δ 87.2. Additional changes were observed for the valine ketone, α-proton, and succinimide methine protons. These data support hydroxylation at the 3-position of the ring, consistent with the structure of moiramide C (**3**), which is hydroxylated at the same site. The similarity in chemical shifts across this moiety suggests an identical relative configuration to that found in moiramide C (Table S7).

### 2.3 Proposed Biosynthesis of Butuanimides

The 2052S draft genome was previously sequenced and assembled by the Joint Genome Institute and deposited in the Integrated Microbial Genomes & Microbiomes (IMG) database with accession number 2541046951. A previous analysis using antiSMASH (v7.0) revealed four conserved BGCs in the PKS and NRPS class.^21^ One PKS-NRPS hybrid cluster included several proteins with similarity to those involved in andrimid biosynthesis encoded by the *adm* locus, including BtmJ (AdmC), BtmL (AdmK), BtmN (AdmL), BtmO (AdmM), BtmP (AdmN), BtmQ (AdmO), and BtmR (AdmP)—PKS and NRPS enzymes involved in the incorporation of the Val-succinimide fragment in the andrimid pathway.^31^ Additionally, we noted the presence of a PAL homologue, BtmG, which would be required to generate the proposed starter unit for biosynthesis. BtmG is 32% identical to AdmH, which, in andrimid biosynthesis, makes the β-Phe residue. This led us to propose the *btm* gene cluster for butuanimide biosynthesis (Figure 4). Though several attempts to generate in-frame, scarless deletions of biosynthetic enzymes were unsuccessful, an insertion disruption mutant of *btmB* resulted in a loss of compound production (Figure S42).

**Figure 4:**
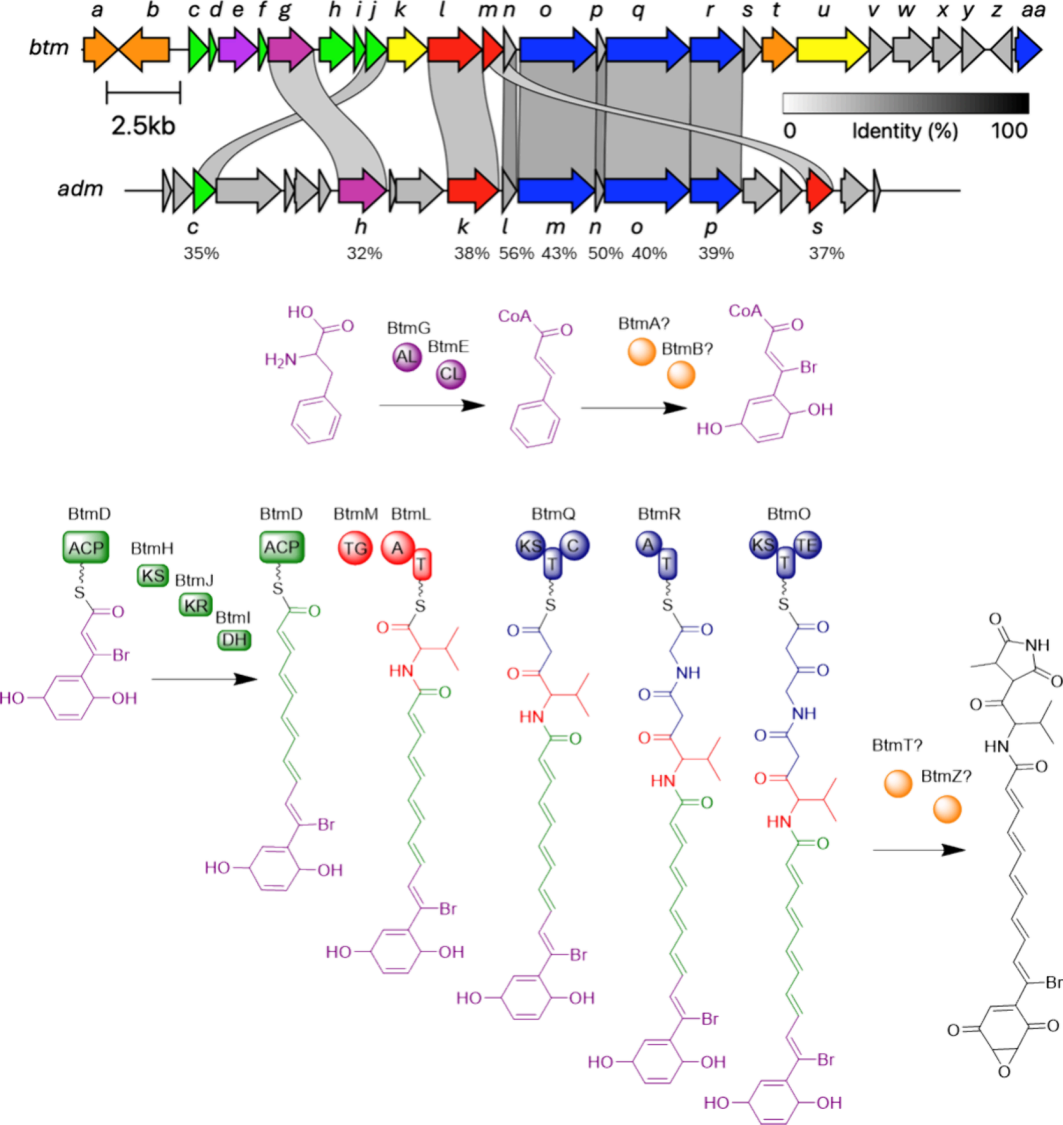
Proposed biosynthesis of butuanimide A (**8**) and comparison of *btm* with *adm* from andrimid biosynthesis (GenBank AY192157). Pathway map made with clinker.^34^ Genes are color coded to the following predicted enzymes: Blue: succinimide-assembling PKS-NRPS; Green: polyene-synthesizing type II PKS (andrimid) or arylpolyene (butuanimide) PKS; Orange: oxidative or other tailoring enzymes; Purple: phenylalanine-modifying starter (butuanimide) or extender (andrimid) synthesis; Red: Valine-adding NRPS and transglutaminase (TG)-like; Yellow: efflux transporters. Percent identity between *btm* and *adm* homologues is indicated below the respective *adm* gene. Domain annotations: ammonia lyase (AL), CoA ligase (CL), acyl carrier protein (ACP), ketosynthase (KS), ketoreductase (KR), dehydratase (DH), adenylation (A), thioesterase (TE), thiolation/peptidyl carrier protein (T).

AdmKMOPS proteins build andrimid’s Val-succinimide warhead moiety, which is essential for biological activity. The presence of similar genes in andrimid-like BGCs and in *btm* was congruent with the butuanimide structures. Further, this similarity suggested that compounds **1**, **2**, and **8** **–** **12** all have the same absolute configuration in the warhead (green) moiety shown in Figure 1, which is supported by NMR chemical shift comparisons. Although both *btm* and *adm* encoded a similar PAL, *btm* completely lacked genes for incorporating β-Phe (*admI* and *admJ*), consistent with the lack of that extender unit in butuanimides. Instead, the *btm* genes were consistent with the use of a Phe derivative as the starter unit. This represents an interesting potential repurposing of a biosynthetic motif to create two quite different chemistries.

Both the andrimid and butuanimide polyketide chains are likely made via type II PKSs,^32,33^ but the proteins were not similar and appeared to have been recruited from different starting points. antiSMASH identified the proteins BtmD and H-J as being arylpolyene-type, consistent with the structural variation found in the pathway. Four additional oxidative *btm* enzymes were present that we proposed to introduce the epoxyquinone functional groups and halogen substituent (*btmA, btmB, btmT,* and *btmZ)*. The mechanistic order of oxidation and halogenation remains uncertain, and it is currently unknown which enzyme is responsible for each specific reaction. Overall, comparison of *btm* and *adm* pathway architecture suggested that these pathways evolved via recombination to generate different carbon skeletons that both incorporate identical Val-succinimide warheads. Several natural product classes contain epoxyquinone fragments, but their biosynthetic enzymes appear to be nonhomologous with those for butuanimide.

*btm* was identified as gene cluster family (GCF) GCF_122 in our previous work.^21^ Many GCFs from shipworm isolates are widely distributed in the animals worldwide and are found in multiple isolates. However, like the majority of GCFs so far identified, GCF_122 was a singleton that we have so far only identified in one *Teredinibacter* strain. This further reinforces the richness of shipworm symbionts as sources of antibacterial agents with novel chemical architectures.

### 2.4 Biological Activity of Butuanimides

Butuanimides **8** **–** **12** purified from 2052S as described were tested for antimicrobial and cytotoxic activity. It is noted that compound instability, particularly at high concentrations, made biological screening difficult. Compounds **8** **–** **10** exhibited moderate antibacterial activity against *Staphylococcus aureus* (MIC_90_ 4–8 μg/mL) and *Enterococcus faecium* (MIC_90_ 8–16 μg/mL), without significant activity against Gram-negative strains (Table 1). Interestingly, the chlorinated analog **9** was significantly more cytotoxic against HEK293 cells (IC_50_ = 0.3–0.9 μg/mL) than both the brominated **8** and **10**, indicating that halogen identity is important for influencing eukaryotic cell toxicity, but not antibacterial potency. This might be due to the greater stability of the Cl derivative compared to the Br forms. (Note: different values, rather than averages, are provided for different cytotoxicity screening biological replicates because of the longer time-course of those assays, during which some amount of the natural products may degrade.)

Andrimid (**1**) and its structural analog moiramide B (**2**) block bacterial AccAD, part of the ACC complex comprising AccA-D that is essential to fatty acid biosynthesis. In andrimid-producing strains, the *accD* gene is duplicated, and a homologue that confers self-resistance (*admT*) is found in the *adm* cluster.^35^ A cocrystal structure of the *S. aureus* ACC CT bound to moiramide B (**2**) shows this interaction is mediated through the enol–enolate form of the diketone moiety, which is anionic and binds to the active site as a competitive inhibitor (Figure 5).^9^ The presence of the β-phenylalanine (absent in butuanimides) led to a 15% improvement in inhibition but did not make close interactions with the enzyme. Butuanimides have the identical diketone warhead, suggesting that they might also block ACC, but notably there is no duplicated ACC gene in the 2052S genome. *accB* and *accC* are found a few open reading frames away from the *btm* cluster, but they are not duplicated elsewhere, and they are different subunits than the AccAD that binds the andrimid warhead. Therefore, we aimed to determine whether ACC is a target of butuanimides.

**Figure 5:**
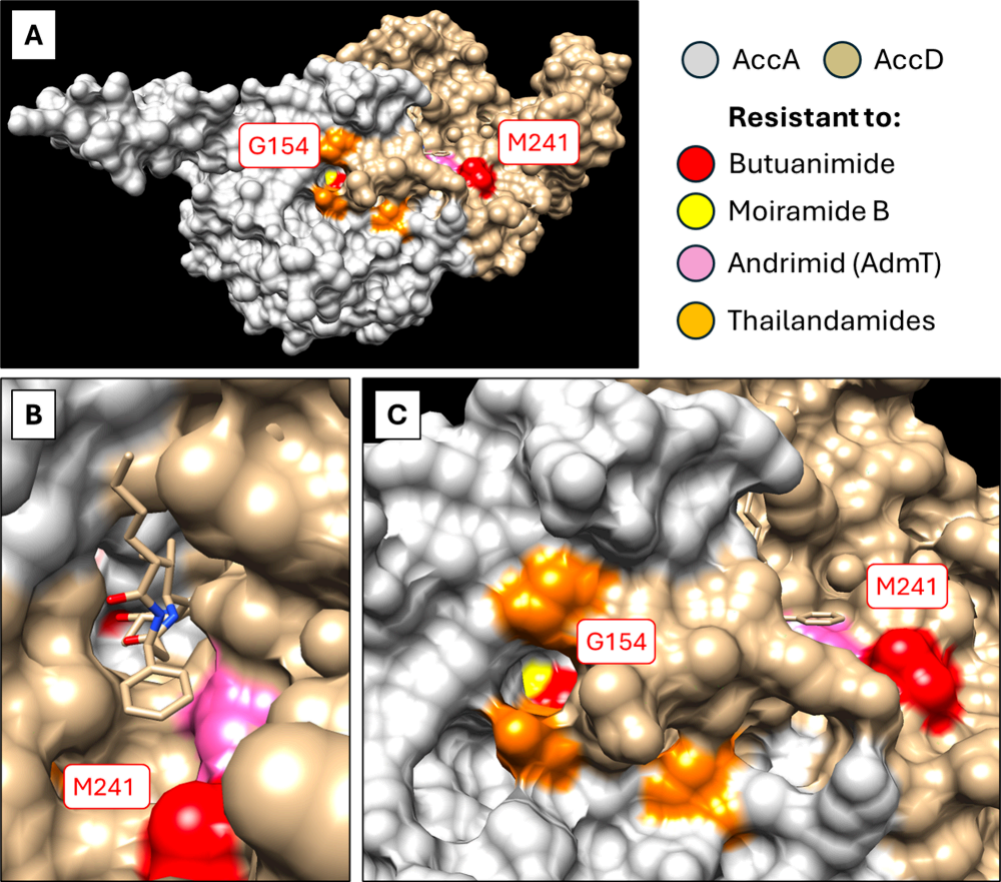
Acetyl CoA carboxylase (ACC) mutations conferring butuanimide resistance. (A) The moiramide B (**2**) (stick figure) cocrystal structure^9^ with AccA (gray) and AccD (brown) was modified using ChimeraX.^37^ AccA and AccD subunits are shown in gray and brown, respectively. Resistance mutations identified following *A. baylyi* treatment with butuanimide are shown and labeled in red. Mutations associated with moiramide/andrimid resistance are shown in yellow and pink, while mutations associated with thailandamides are shown in orange. (B) Side view highlighting the AccA/D interface region containing clustered moiramide and butuanimide resistance mutations, consistent with a shared binding region proximal to the acetyl-CoA entry site; the G154S mutation is shown at the rear of this region. (C) Enlarged view of panel A illustrating that moiramide and butuanimide resistance mutations localize along a continuous channel at the AccA/D interface where acetyl-CoA normally binds, whereas thailandamide resistance mutations localize to a distinct region overlapping the known biotin-binding site. The benzyl moiety of moiramide B (**2**) is shown for reference and does not extend to the M241T residue; instead, the side chain projects along a CoA-binding channel. This spatial separation suggests possible divergence of moiramide and butuanimide interactions within AccD while maintaining a common entry region. Note: This figure maps experimentally identified resistance mutations onto a static crystal structure and does not imply a unique binding pose or ligand trajectory beyond the spatial constraints imposed by the mutation distribution.

An antibiotic sensitive strain of *Acinetobacter baylyi* was used to generate mutant strains resistant to butuanimides due to the well-developed genetic tools available for the strain.^36^ This strain is efflux deficient (Δ*adeIJK*) and has a compromised outer membrane (Δ*lptE*) to ensure that the compound is able to enter the cell and reach its molecular target. Due to the difficulty separating the chlorinated and brominated analogs at a sufficient scale, a mixture of approximately 66% **8** and 34% **9** was used in this experiment (as determined by ^1^H NMR integration of H-22, Figure S41). This mixture had an MIC_90_ of 8 μg/mL in the sensitive strain. Site-directed mutagenesis in the genomic region of the ACC genes resulted in the generation of several strains resistant to the butuanimides. Strikingly, 12 mutants arose with an identical mutation in AccA (G154S) (Figure 5). These strains were validated with broth microdilution assays and displayed an 8-fold increase in MIC. This mutation is within the active site region of AccA, strongly supporting this protein as the molecular target in bacteria. The same mutation was previously identified in moiramide B (**2**) resistance in *Staphylococcus aureus* and *Bacillus subtilis*, indicating a widely shared binding mechanism.^2^ In the same study, it was demonstrated that moiramide B (**2**) is a competitive inhibitor of the (CT) site, a finding later replicated with andrimid.^9^ Thailandamide, another ACC inhibitor, is also blocked by mutations in the same region,^5^ in addition to other mutations in AccA found in *Salmonella enterica*.^4^ An additional 4 *A. baylyi* M241T mutations were discovered in the AccD active site, which conferred less robust resistance of 2X MIC in broth assays. Similarly, moiramide B (**2**) induced an AccD resistant mutant in *Escherichia coli*, although not in the same position as found in *A. baylyi*.^2,10^ An AccD mutation is also present in AdmT, an AccD analog that confers resistance to moiramide B (**2**).^35^

Using the previous cocrystal structure of AccAD with moiramide B (**2**) (Protein Data Bank, 5KDR) as a model, mutations were mapped, showing that butuanimides bind at a common succinimide warhead pocket in AccA (shown in red, position G154 in Figure 5). The butuanimide-resistant mutation in AccD potentially mapped a different channel from that followed by the polyene portion of **1** and **2** (shown in red, position M241 in Figure 5).

## 3 Conclusions

We describe five butuanimides that are induced by quorum-sensing regulation in shipworm symbiotic bacteria. Compounds **8** **–** **10** are antibacterial against two Gram-positive bacterial species but have insignificant potency against Gram-negative pathogens. Anti-Gram-negative activity could be improved in a strain with a compromised outer membrane, indicating that, as found in related natural products, cell entry is the limiting factor. The measured MICs are roughly in the same order of magnitude as those previously reported for other succinimide antibiotics **1** and **2**, indicating flexibility in the side chain that will be useful in further ACC inhibitory drug design. By contrast, we could not previously detect antibacterial activity in **11** and **12** against the ESKAPE panel, suggesting the possibility of applying redox control to antibiotic activity.

Mutagenesis in *A. baylyi* showed that butuanimides **8** and **9** target AccA and AccD. The AccA mutation is in the same site that binds the moiramide B warhead and thailandamide B, indicating an underlying similarity in mechanism in the CT active site. Additional mutations in AccD suggest that the AccA/D active compounds may have different binding sites, affording opportunities for further drug design. It has been speculated that such differences may be at least in part due to the compounds altering the channels normally occupied by biotin and/or coenzyme A.^4^ It is notable that, in contrast to andrimid-producing strains, the target *acc* genes are not duplicated. We were only able to isolate the active molecules through sequestration on hydrophobic resin, while the inactive hydroquinones were isolated without added resin. This suggests that 2052S controls self-resistance through the oxidation state of the expoxyquinone moiety, rather than target gene duplication. Additionally, the presence of a nearby AccB/C-encoding locus suggests the possibility of an altered interface with AccA/D that could potentially confer resistance. Recently, the active heteromeric form of bacterial ACC has been elucidated by cryo-EM,^38^ but the complex nature of this multiprotein assembly makes it difficult to assign potential interface residues with certainty.

The preference of many AccA/D inhibitors for Gram-positive bacteria is well documented. For example, natural products **1**, **2**, **4**, and **5** and **8** **–** **10** are all effective against Gram-positive, with much lower activity against Gram-negative bacteria. Interestingly, thailandamide B (**5**) is active against Gram-negative *S. enterica* at 15 μM, marking a relatively active anti-Gram-negative natural product. By contrast, thailandamide A (**4**) was not active except in *E. coli* in which the lipopolysaccharide transporter gene *lptD* was mutated. A similar story unfolds with the butuanimides, which are more active in Gram-positive strains. They were weakly active in Gram-negative *A. baylyi*, but displayed a 4X lower MIC after disruption of the efflux system and outer membrane through disruption of the *lptE* gene. Similarly, the antibiotic efficacy of compound **2** and its synthetic analogs is strongly correlated with their ability to penetrate membranes or to avoid efflux.^12,39^

The presence of epoxyquinone-like motifs in **8** **–** **12** suggests a potential second mechanism of action, or that it might be possible to construct dual-acting derivatives. There are many examples of natural products containing the epoxyquinone and related groups.^40,41^ At least one such compound binds covalently to its molecular target,^42^ indicating that the epoxyquinone in **8** and **9** might have a second target. For example, we still do not know the molecular basis of mammalian cell cytotoxicity for these compounds. Other limitations of this study are also derived from the relative instability of epoxyquinone/quinol. The active oxidized forms **8–10** could only be isolated using resin, and were otherwise unstable, while the seemingly inactive reduced forms **11** **–** **12** were only isolated absent resin. In addition, many peaks in HPLC chromatograms were clearly visible, indicating a large family of related compounds in small amounts, which we believe are degradation products of the active natural products. For this reason, we could not determine an absolute configuration for the epoxyquinone fragment. Modest differences in IC_50_ measurements in biological replicates in the longest assay (cytotoxicity) reflected instability over extended incubation. In highly concentrated samples of **8**, degradation in DMSO led mainly to an undefined, blackish insoluble residue; this process was greatly accelerated through freeze–thaw cycles or drying and resuspending the sample, and the only obvious molecule still present in solution by MS after degradation matched the mass of **8** with the addition of HBr (Figure S40). Metastability of natural products is a common issue in the field that is often viewed as a nuisance. Here, metastability is potentially a benefit, as the reduced forms **11** and **12** are inactive against bacteria. Potentially, their oxidative modification into **8–10** controls their biological activity. This might prove to be a useful quality, as several approved antibiotics are active only in low-oxygen conditions, leading to a lower incidence of resistant mutations.^43–45^

Notably, andrimid-like compounds have been isolated from several host-associated bacterial strains.^39,46^ Andrimid itself was originally discovered from an *Enterobacter sp.* associated with insect eggs.^3^ The identification of butuanimides from a shipworm endosymbiont, together with their quorum-sensing-regulated production, suggests a recurring association between this class of ACC inhibitors and symbiotic lifestyles. While their ecological function has not been directly established, their activity against Gram-positive bacteria is consistent with a potential role in mediating microbial competition or contributing to host-associated defensive chemistry. Alternatively, if produced at low levels in the host, such compounds could contribute to microbial communication, as the ACC complex is often a regulatory hub in various organisms.^47–49^

In summary, here we expand the structure–activity relationships of antibacterial ACC inhibitors, a group of lead antibiotics, providing a basis for further compound development of this important natural product structural class.

## Supplementary Material



## Notes

Experimental methods and supplementary figures: NMR, HR-MS, and gene disruption analysis; supplementary tables: tabulated NMR data, BLAST results for biosynthetic genes, and strains, plasmids, and primers used in this study (PDF)

## ∥

Department of Biomedical and Pharmaceutical Sciences, University of Rhode Island, Kingston, Rhode Island 02881, United States

## ⊥

Department of Microbiology, Cornell University, Ithaca, New York 14853, United States.

## #

Department of Chemistry, College of Science, De La Salle University, Manila 1004, Philippines.

## ∇

Department of Medicinal Chemistry, University of Utah, Salt Lake City, Utah 84112, United States.

## ○

Philippine Genome Center, University of the Philippines, Diliman, Quezon City 1101, Philippines.

## ◆

Department of Chemistry, University of Utah, Salt Lake City, Utah 84112, United States.

## ¶

Division of Genetics, Oregon National Primate Research Center, Oregon Health and Science University, Beaverton, Oregon 97006, United States.
