# Supplementary material for: Butuanimides, Fatty Acid Synthesis-Inhibiting Antibiotics from Symbiotic Bacteria
Source: ACS Chem Biol. 2026 Apr 28;21(5):1125–34. doi: 10.1021/acschembio.6c00130 (PMC13151354; doi:10.1021/acschembio.6c00130)
Supplement: Supplementary file 1 [file cb6c00130_si_001.pdf]

Supplemental Information

Butuanimides, Fatty Acid Synthesis-Inhibiting Antibiotics from Symbiotic Bacteria

Bailey W. Miller, [1†] *Albebson L. Lim,*[1†] *Jeannie Bailey,*[2] *Mark Jeremiah B. Cleofas,*[3†] *Noel Lacerna II,*[3†] *Marvin A. Altamia,*[3†] *Jared T. Seale,* [1] *Jose Miguel D. Robes,*[3†] *Hiroaki Naka,*[1†] *Colin Manoil,*[2] *Margo G. Haygood,*[1] *Eric W. Schmidt,*[1*] *and Gisela P. Concepcion,*[3*]

[1]Department of Medicinal Chemistry, University of Utah, Salt Lake City, UT 84112, USA

[2]Department of Genome Sciences, University of Washington, Seattle, WA 98195, USA.

[3]The Marine Science Institute, University of the Philippines, Diliman, Quezon City 1101, Philippines

Table of Contents

Experimental Methods

Supplemental Figures

Figure S1. HRESIMS of compound **8**
Figure S2. ^{1}H NMR spectrum of **8** in DMSO-d_6 (500 MHz)
Figure S3. ^{13}C NMR spectrum of **8** in DMSO-d_6 (125 MHz)
Figure S4. gCOSY NMR spectrum of **8** in DMSO-d_6 (500 MHz)
Figure S5. zTOCSY NMR spectrum of **8** in DMSO-d_6 (500 MHz)
Figure S6. gHSQCAD NMR spectrum of **8** in DMSO-d_6 (500 MHz)
Figure S7. gHMBCAD NMR spectrum of **8** in DMSO-d_6 (500 MHz)
Figure S8. NOESY NMR spectrum of **8** in DMSO-d_6 (500 MHz)
Figure S9. HRESIMS of compound **9**
Figure S10. ^{1}H NMR spectrum of **9** in DMSO-d_6 (500 MHz)
Figure S11. ^{13}C NMR spectrum of **9** in DMSO-d_6 (125 MHz)
Figure S12. ^{13}C NMR comparison of **8, 9,** and a mixture of **8** and **9** in DMSO-d_6 (125 MHz)
Figure S13. gCOSY NMR spectrum of **9** in DMSO-d_6 (500 MHz)
Figure S14. zTOCSY NMR spectrum of **9** in DMSO-d_6 (500 MHz)
Figure S15. gHSQCAD NMR spectrum of **9** in DMSO-d_6 (500 MHz)
Figure S16. gHMBCAD NMR spectrum of **9** in DMSO-d_6 (500 MHz)
Figure S17. NOESY NMR spectrum of **9** in DMSO-d_6 (500 MHz)
Figure S18. HRESIMS of compound **10**
Figure S19. ^{1}H NMR spectrum of **10** in DMSO-d_6 (500 MHz)
Figure S20. gCOSY NMR spectrum of **10** in DMSO-d_6 (500 MHz)
Figure S21. zTOCSY NMR spectrum of **10** in DMSO-d_6 (500 MHz)
Figure S22. gHSQCAD NMR spectrum of **10** in DMSO-d_6 (500 MHz)
Figure S23. gHMBCAD NMR spectrum of **10** in DMSO-d_6 (500 MHz)
Figure S24. NOESY NMR spectrum of **10** in DMSO-d_6 (500 MHz)
Figure S25. HRESIMS of compound **11**
Figure S26. ^{1}H NMR spectrum of **11** in DMSO-d_6 (500 MHz)
Figure S27. gCOSY NMR spectrum of **11** in DMSO-d_6 (500 MHz)
Figure S28. zTOCSY NMR spectrum of **11** in DMSO-d_6 (500 MHz)
Figure S29. gHSQCAD NMR spectrum of **11** in DMSO-d_6 (500 MHz)
Figure S30. gHMBCAD (5 Hz coupling) spectrum of **11** in DMSO-d_6 (500 MHz)
Figure S31. gHMBCAD (10 Hz coupling) spectrum of **11** in DMSO-d_6 (500 MHz)
Figure S32. NOESY NMR spectrum of **11** in DMSO-d_6 (500 MHz)
Figure S33. HRESIMS of compound **12**
Figure S34. ^{1}H NMR spectrum of **12** in DMSO-d_6 (500 MHz)
Figure S35. ^{13}C NMR spectrum of **12** in DMSO-d_6 (125 MHz)
Figure S36. gCOSY NMR spectrum of **12** in DMSO-d_6 (500 MHz)
Figure S37. zTOCSY NMR spectrum of **12** in DMSO-d_6 (500 MHz)
Figure S38. gHSQCAD NMR spectrum of **12** in DMSO-d_6 (500 MHz)
Figure S39. gHMBCAD NMR spectrum of **12** in DMSO-d_6 (500 MHz)
Figure S40. HRMS spectrum of recovered degradation product of **8**
Figure S41. Gene disruption of *btmB* ablates production of butuanimides.
Figure S42. ^{1}H NMR spectrum of mixture of **8** and **9** used in bioassays.

Supplemental Tables

Table S1. ^{13}C and ^{1}H NMR chemical shift comparisons between compound **8** and andrimid (**1**).
Table S2. NMR data for compound **8**
Table S3. NMR data for compound **9**
Table S4. NMR data for compound **10**
Table S5. NMR data for compound **11**
Table S6. NMR data for compound **12**
Table S7. Comparison of ^{1}H and ^{13}C NMR chemical shifts of **12** and **3**.
Table S8. Annotations of gene in *btm* biosynthetic gene cluster, including identity comparison to nearest *adm* gene homologs.
Table S9. Strains and plasmids used in this study
Table S10. Cloning and diagnostic primers used in this study

References

EXPERIMENTAL METHODS

General Experimental Procedures. High-resolution mass spectra were obtained on a Waters Xevo G2-XS QTof mass spectrometer equipped with a Zspray ESI source. Chromatography for LC-MS was performed using an Aquity H class UPLC system with a Waters Acquity HSS T3 column (2.1x100mm, 1.8 μm). NMR data were collected using a Varian 500 MHz NMR spectrometer with a 5 mm Varian HCN Oneprobe (^{1}H 500 MHz, ^{13}C 125 MHz). Residual signals from solvents were used for referencing. HPLC was performed using a Dionex Thermo UltiMate 3000 system equipped with an autosampler and diode array detector. Analytical HPLC was performed using a Phenomenex Luna C18 column (4.6x150mm, 5μm) and semi-preparative HPLC was performed using a Phenomenex PhenylHexyl column (10x250mm, 10μm). Flash chromatography was performed on a Teledyne CombiFlash NextGen 100 system.

T. turnerae **Growth and Compound Isolation**. *T. turnerae* 2052S.S.stab0a.01 was revived from glycerol stocks on solid shipworm basal medium (SBM) agar supplemented with 0.2% cellulose,[1] then incubated at 30ºC until distinct colonies formed. Individual colonies were excised from the agar and used to inoculate 50 mL liquid SBM cultures, which were incubated for 2 days with shaking at 150 rpm at 25ºC. These seed cultures were used to inoculate 1 L of SBM broth with 0.3% cellulose in 2.8 L Fernbach flasks.

For compounds **11** and **12**, 4 flasks were incubated for 5 days, then clarified by centrifugation (ThermoBioFlex rotor, 5,500 rpm, 4ºC) to provide supernatant for the subsequent steps. The supernatant was incubated with Diaion HP-20 resin, which was collected by filtration over clean cotton, washed with water, then eluted in a step gradient of 25%, 50%, and 100% methanol in water. The 50% and 100% methanol fractions were combined and subjected to C_{18} open column reversed phase chromatography to produce eight subfractions (10%, 20%, 30%, 40%, 50%, 75%, 100% MeOH, 1:1 DCM/MeOH). The subfraction eluting with 75% methanol in water was further purified by semi-preparative HPLC (Phenomenex Luna C_{18}, 10mm x 250mm, 3.0 mL/min, 35%

MeOH in H_2O), and two peaks were collected resulting in compounds **11** (t_R=15 min, 0.73 mg) and **12** (t_R=18 min, 2.71 mg). Subsequent batches were grown to obtain additional material for NMR studies.

For compounds **8-10**, 6 Fernbach flasks (1 L SBM each) were inoculated with 1 mL each of the seed culture. After 24 hours, Diaion HP-20 resin (20 mL) was added as a slurry in neat ethanol, then the cultures were incubated for 4 days. During this time, the culture broth and cellular debris turned brown, while the resin was stained a deep red. Resin was collected by filtration through Millipore MiraCloth, then washed with water and eluted in a step gradient of 25%, 50%, 75%, and 100% acetonitrile in water. The 75% and 100% acetonitrile fractions were combined and dried in vacuo. These fractions were resuspended in acetonitrile and a portion was purified by semi-preparative HPLC (Phenomenex Luna PhenylHexyl, 10mm x 250 mm, 4.0 mL/min, 55% ACN) to yield three compounds: **8** (t_R=8.3 min, 2.1 mg), **9** (t_R=14.3 min, 0.8 mg), and **10** (t_R=15.25 min, 5.1 mg).

To obtain the material for mutagenesis studies, strain 2052S was grown in SBM (14 L) with resin as described above. The 100% ACN elution was subjected to flash chromatography with a 100g C_{18} RediSep GOLD column using the following elution gradient: 10% ACN from 0-1 min, linear gradient from 10%-100% ACN from 1-20 min at 60 mL/min flow rate. The major peak (t_R 14 min, 105 mg) was collected and dried in vacuo.

Bioinformatics Analysis. Genomic data for 2052S.S.stab0a.01 was accessed from IMG (Genome ID 2541046951) and analyzed using antiSMASH v7.0. *btm* and *adm* pathways were compared using clinker.[2]

Antibiotic activity screening. MICs were determined through broth microdilution experiments. Glycerol stocks of *E. coli* C600 were revived by streaking on Luria-Bertani agar (LBA) and *Acinetobacter baumannii* (ATCC 19606), *Staphylococcus aureus* (ATCC 1600), *Klebsiella pneumoniae* (ATCC BAA-1705), and *Enterococcus faecalis* (ATCC 29212) on Mueller Hinton II Agar (MHA). Plates were incubated overnight at 37˚C, then single colonies were transferred into

broth and incubated for 6-8 hours at 30˚C with shaking at 150 rpm. The turbidity of the culture was adjusted to match 0.5 McFarland standard ($1x10^8$ cells/mL) then diluted 200-fold and added to each well of a 96-well flat plate. Compounds were added using a two-fold dilution scheme starting at 32 µg/mL. Plates were incubated for 18-20h at 30˚C, 150 rpm, then MTT (10 µL; 5 mg/mL in 1X PBS) was added and incubated for 2 h under the same conditions. The A_{570} was then measured using a Biotek-Synergy 2 Microplate Reader (Biotek).

Mammalian cell antiproliferative assay. HEK-293 (ATCC CRL-1573) cells were grown in RPMI 1640 medium supplemented with 10% fetal bovine serum, 100 units of penicillin, and 100 µg/mL of streptomycin under a humidified environment with 5% CO_2 at 37°C. Cells were seeded in 96-well plates at a density of 10,000 cells per well and incubated for 24 h. Compounds were added in a 2-fold-step serial dilution starting at 32 µg/mL. After 72 h of incubation, the media was removed and MTT (15 µL; 5 mg/mL in 1X PBS) was added to each well. The plates were incubated at 37°C for 3h with 5% CO_2. After incubation, DMSO (100 µL) was added, and absorbance was read at 570nm using a Biotek-Synergy 2 microplate reader (Biotek).

Identification of resistance mutations. Approximately 10-kb PCR fragments carrying *accA* or *accD* were amplified from the genome of *Acinetobacter baylyi* ADP1 with mutagenesis using a low-fidelity polymerase (New England Biolabs LongAmp Taq Polymerase). Fragments were transformed into naturally competent *A. baylyi* ADP1 *ΔadeIJK ΔlptE* and plated on minimal agar containing butuanimides (24-64 µg/ml; ~2-5x MIC) to select resistant mutants. Individual colonies were purified and their *accA* or *accD* genes PCR-amplified and sequenced to identify mutations responsible for resistance.

Gene disruption of *btmB* and analysis of butuanamide production. Gene disruption of *btmB* was achieved via single-crossover insertion mutagenesis into the target locus. To construct the disruption plasmid, an approximately 1.5 kb internal fragment of the btmB gene was amplified by PCR and cloned into the pGEM-T Easy cloning vector, followed by transformation into *E. coli* DH5α λpir. The resulting plasmid was then double-digested with *SpeI* and *SmaI*, and

the insert was ligated into the similarly digested suicide vector pDM4, which carries a kanamycin resistance marker, generating plasmid pDM4_Ins_2366.

The recombinant plasmid was introduced into *E. coli* S17-1 λpir and subsequently transferred into strain 2052S via conjugation. Plasmid cointegrates were selected on SBM agar plates supplemented with 0.5% (w/v) Sigmacell cellulose Type 101 as the sole carbon source and 50 μg/mL kanamycin. Successful disruption mutants were verified by PCR and sequencing.

Both wild-type and *btmB* disruption mutant strains were cultivated under identical conditions. Metabolites were extracted from cultures follow the methods described for compounds **11** and **12** and analyzed by LC–MS to compare butuanimide production.

SUPPLEMENTAL FIGURES

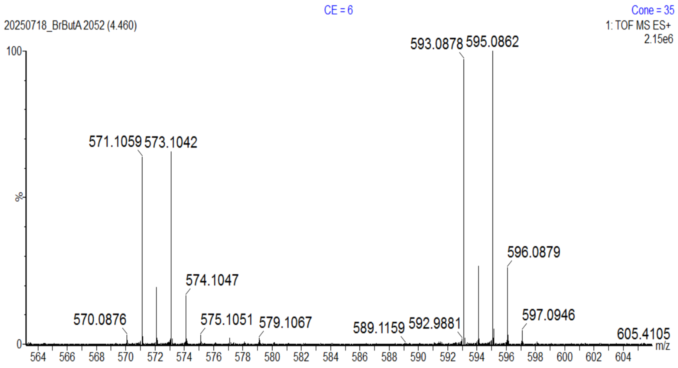

Figure S1. HRESIMS of compound **8**.

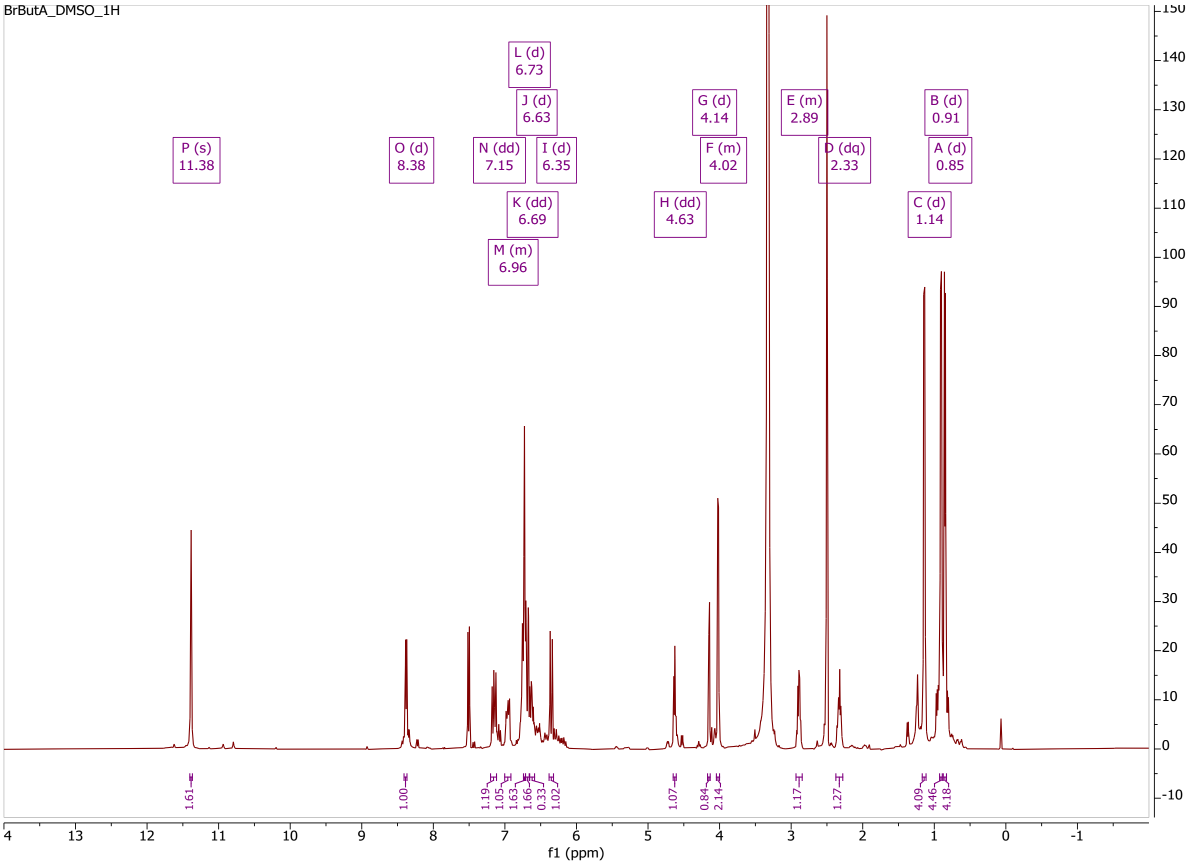

Figure S2. ^{1}H NMR spectrum of **8** in DMSO-d_6 (500 MHz)

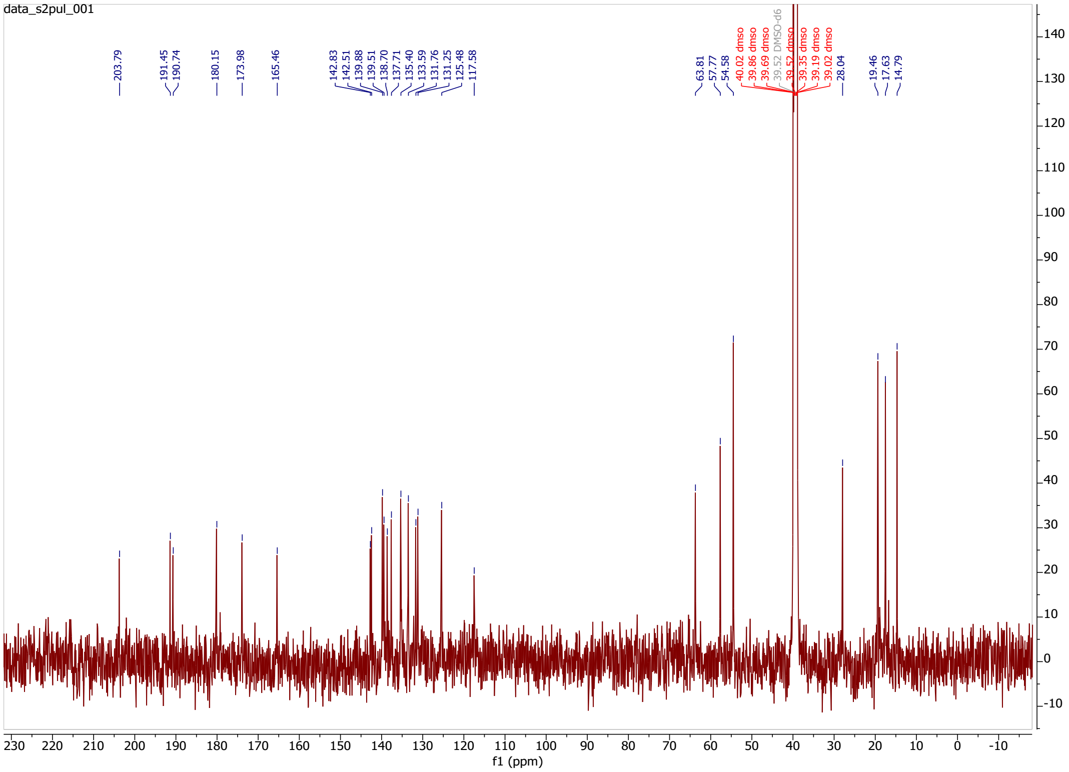

Figure S3. ^{13}C NMR spectrum of **8** in DMSO-d_6 (125 MHz)

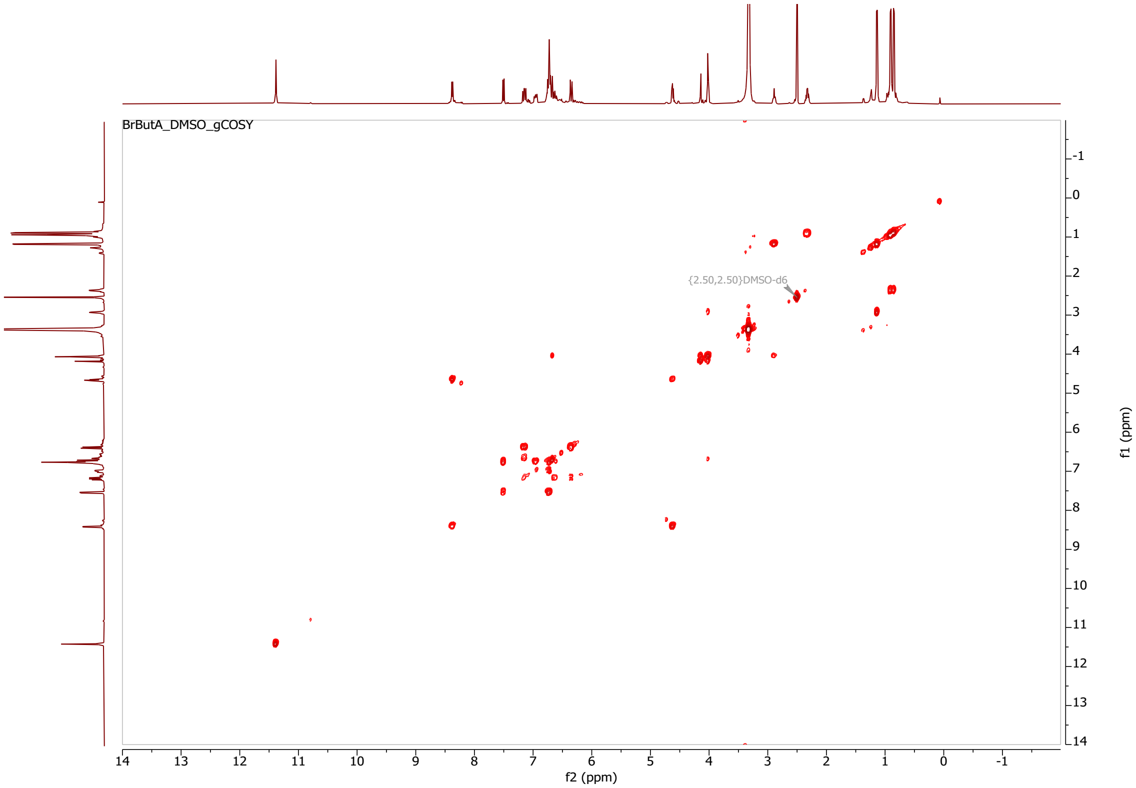

Figure S4. gCOSY NMR spectrum of **8** in DMSO-d_6 (500 MHz)

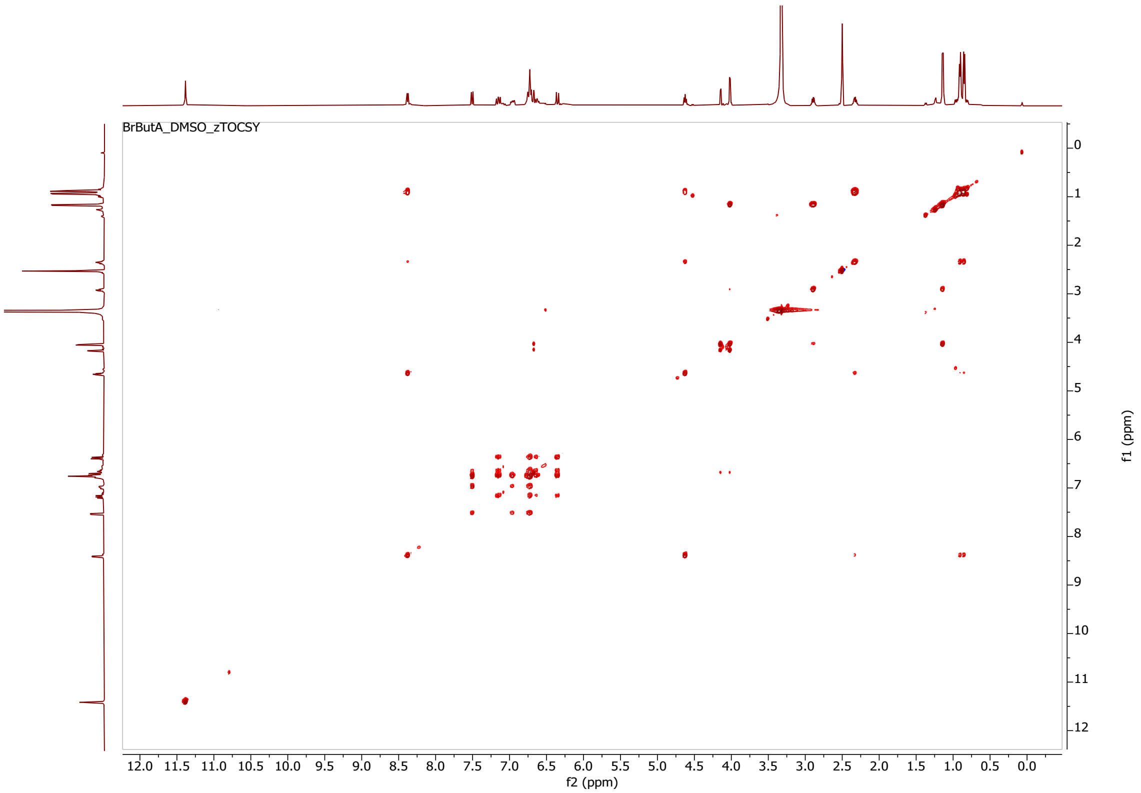

Figure S5. zTOCSY NMR spectrum of **8** in DMSO-d_6 (500 MHz)

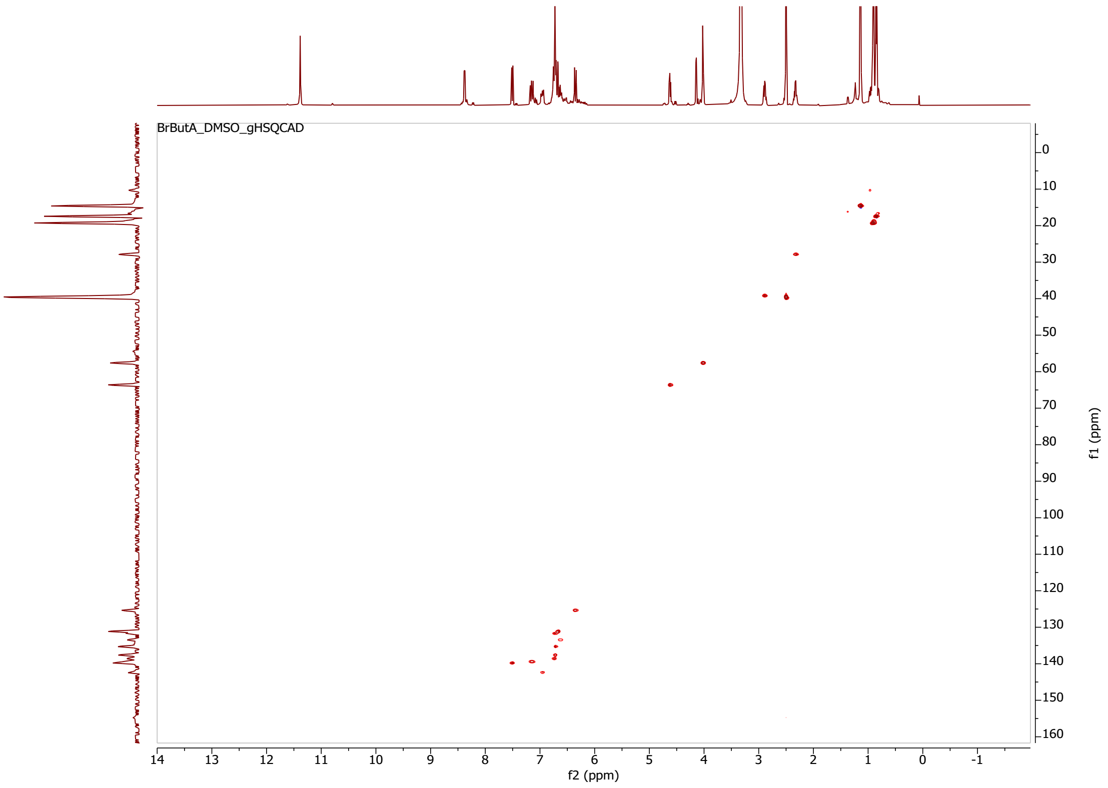

Figure S6. gHSQCAD NMR spectrum of **8** in DMSO-d_6 (500 MHz)

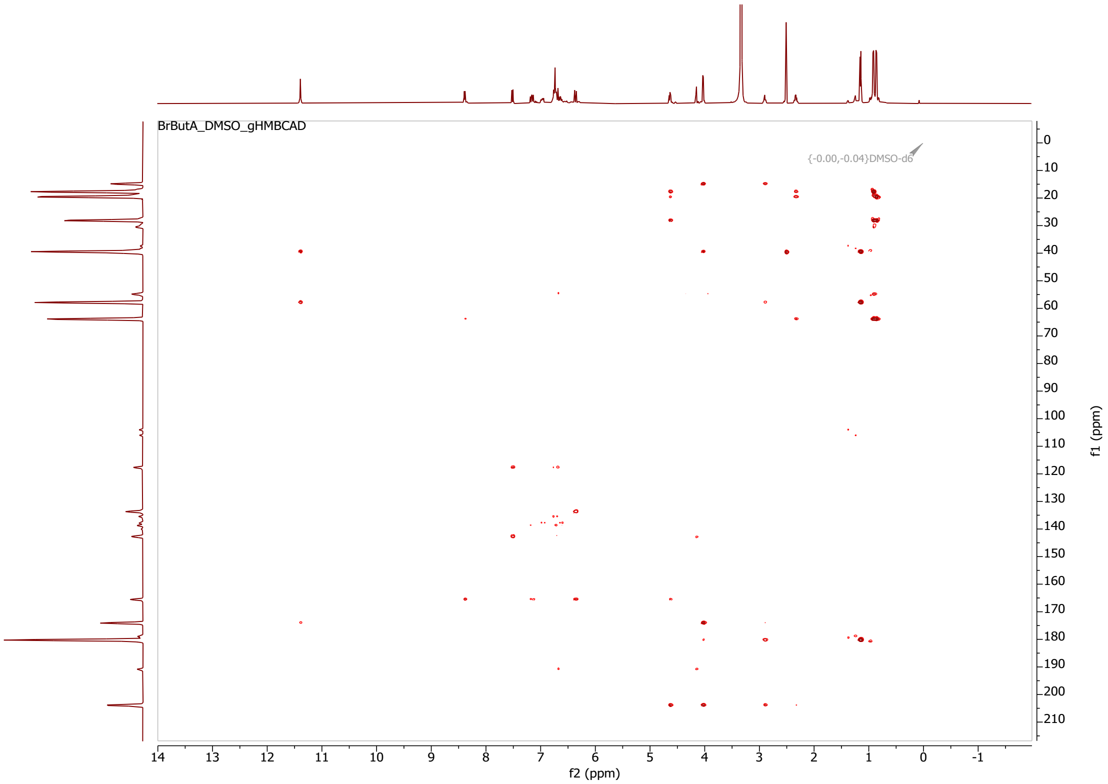

Figure S7. gHMBCAD NMR spectrum of **8** in DMSO-*d*6 (500 MHz)

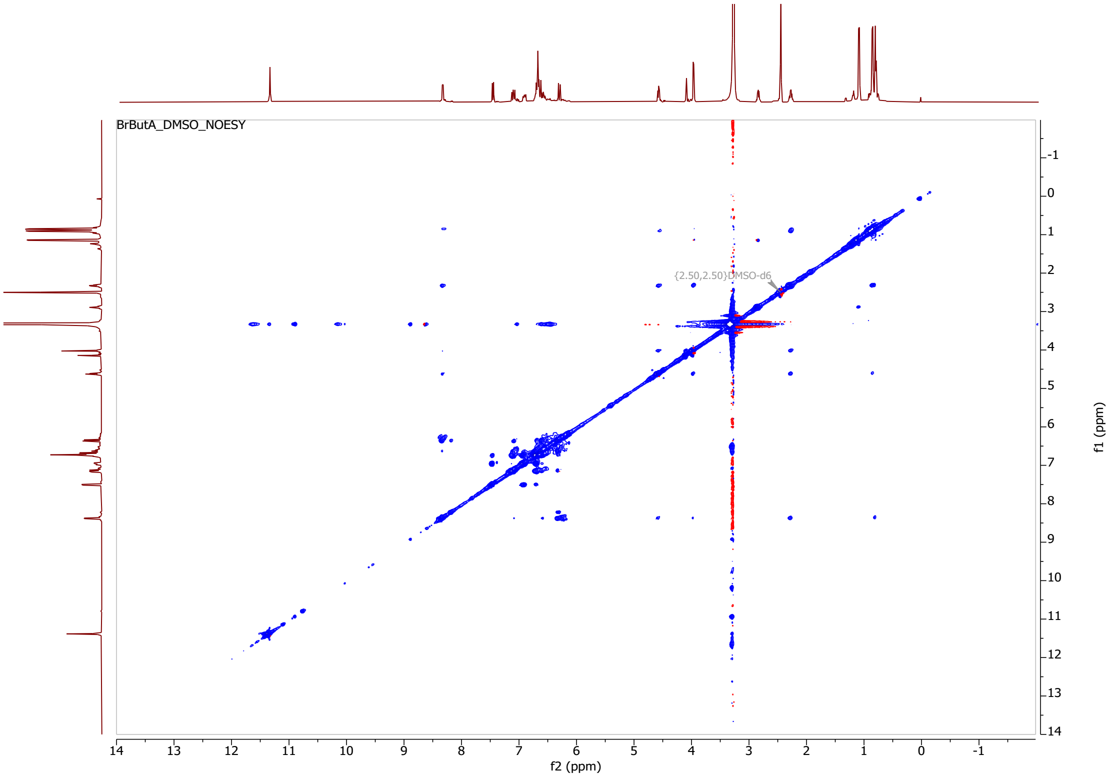

Figure S8. NOESY NMR spectrum of **8** in DMSO-*d*6 (500 MHz)

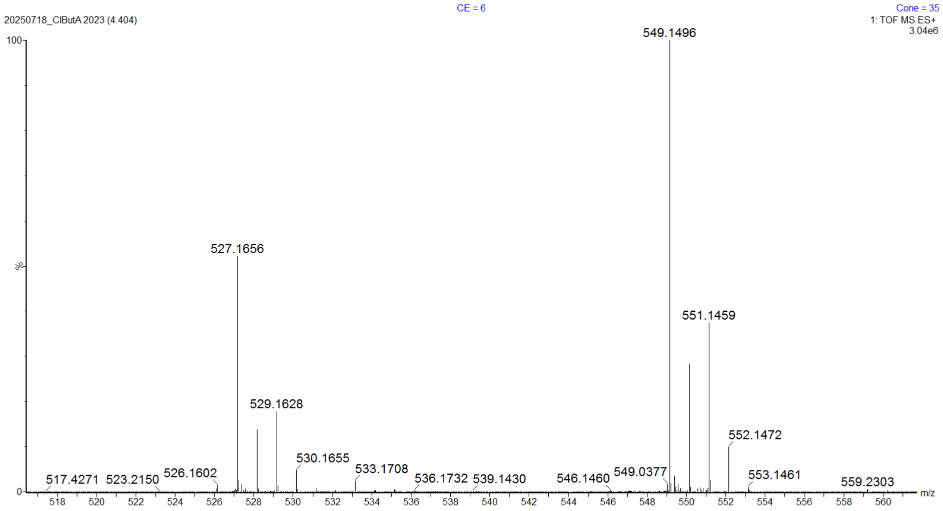

Figure S9. HRESIMS spectrum of **9**

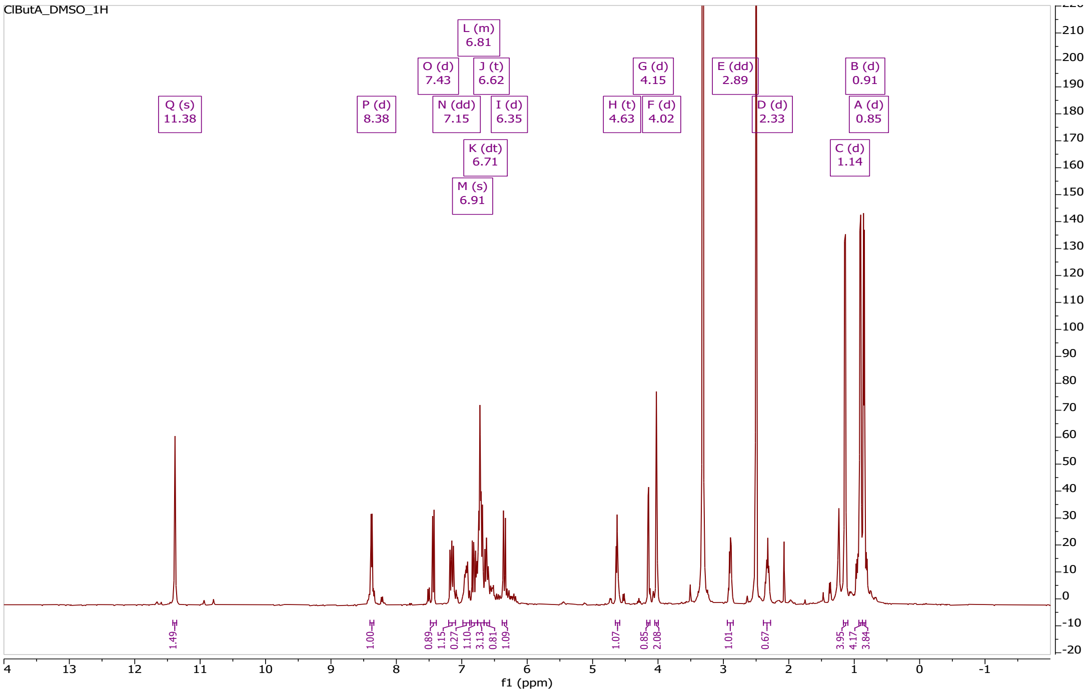

Figure S10. ^{1}H NMR spectrum of **9** in DMSO-d_6 (500 MHz)

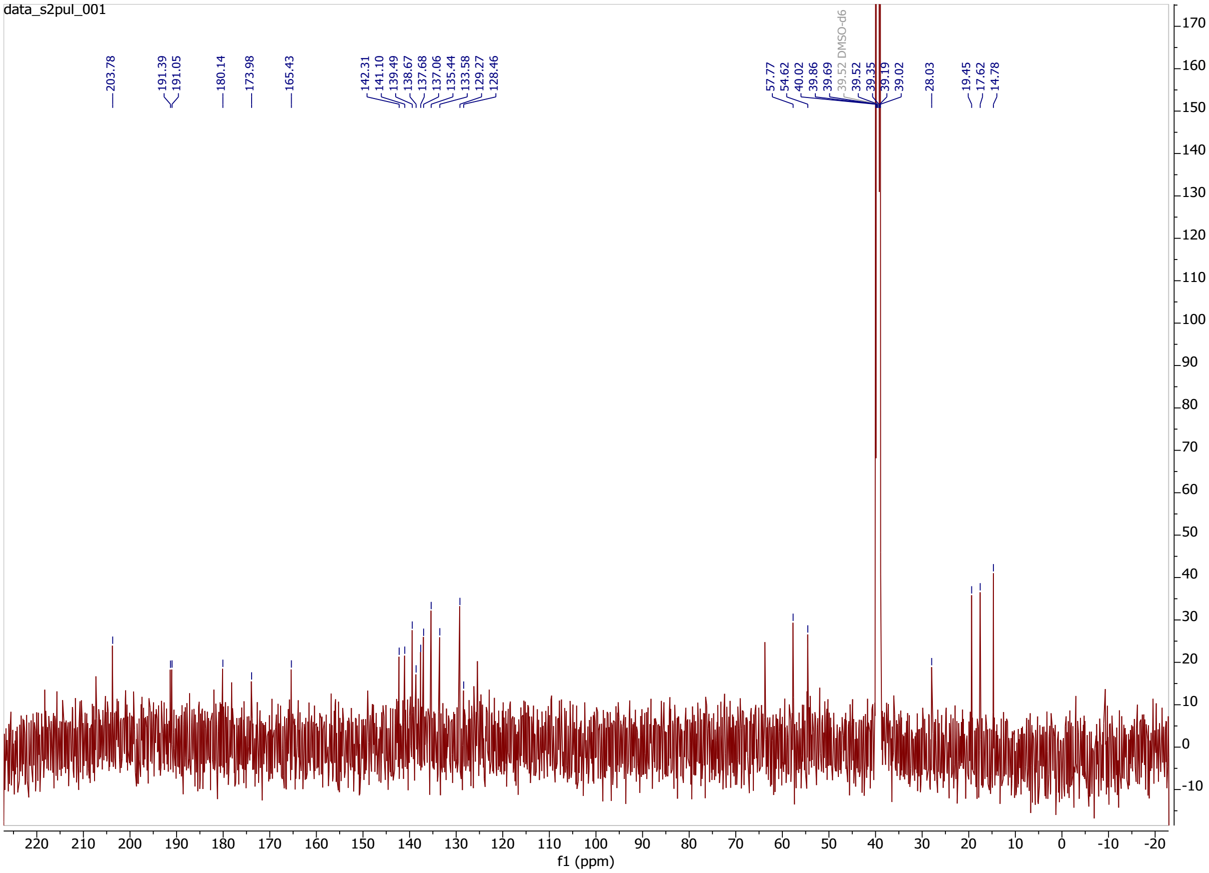

Figure S11. ^{13}C NMR spectrum of **9** in DMSO-d_6 (125 MHz)

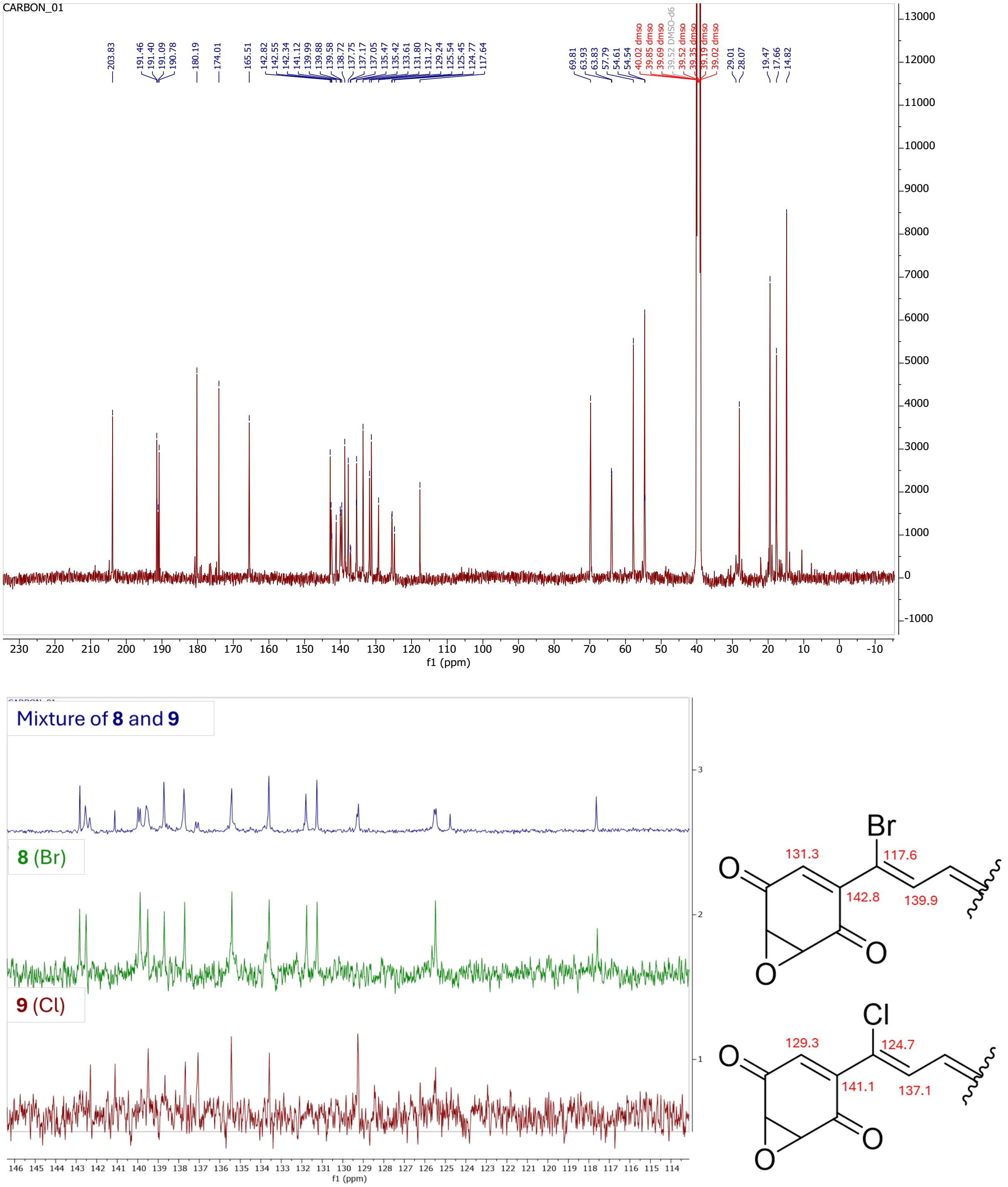

Figure S12. Top: ^{13}C NMR spectrum of a mixture of **8** and **9** in DMSO-d_6 (125 MHz). Bottom: Comparison of olefin region of ^{13}C NMR spectra of pure **8,** pure **9,** and the mixture of **8** and **9** in DMSO-d_6 (125 MHz).

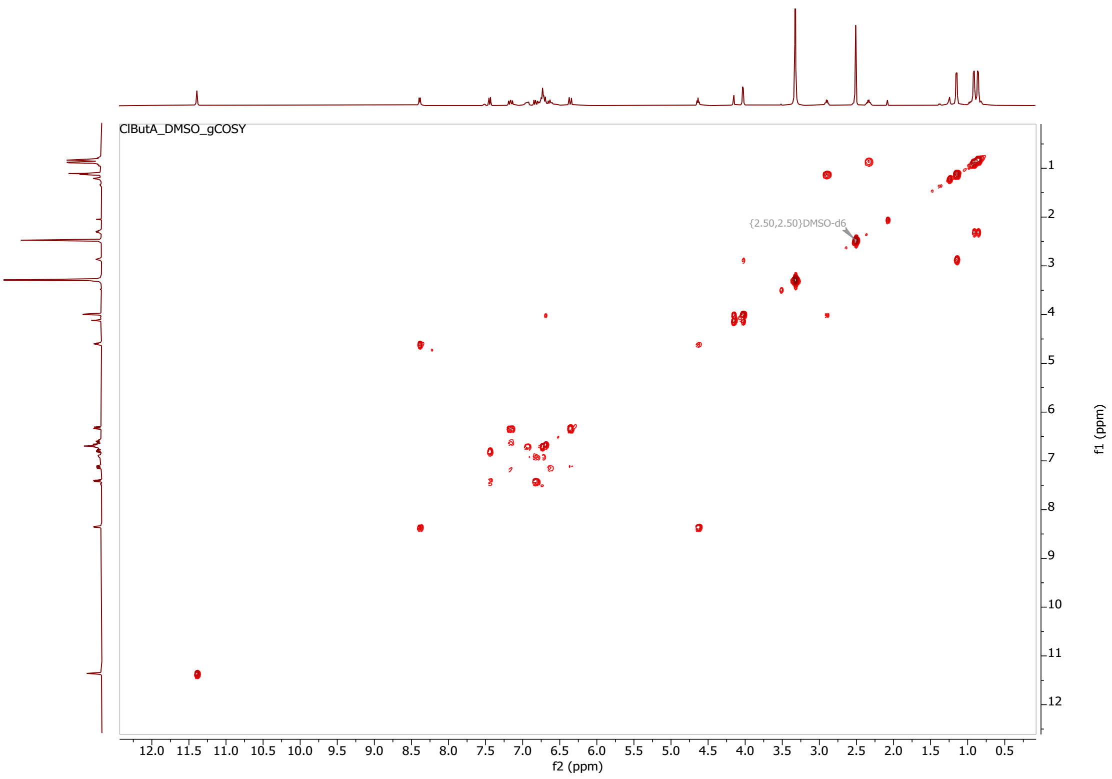

Figure S13. gCOSY spectrum of **9** in DMSO-d_6 (500 MHz)

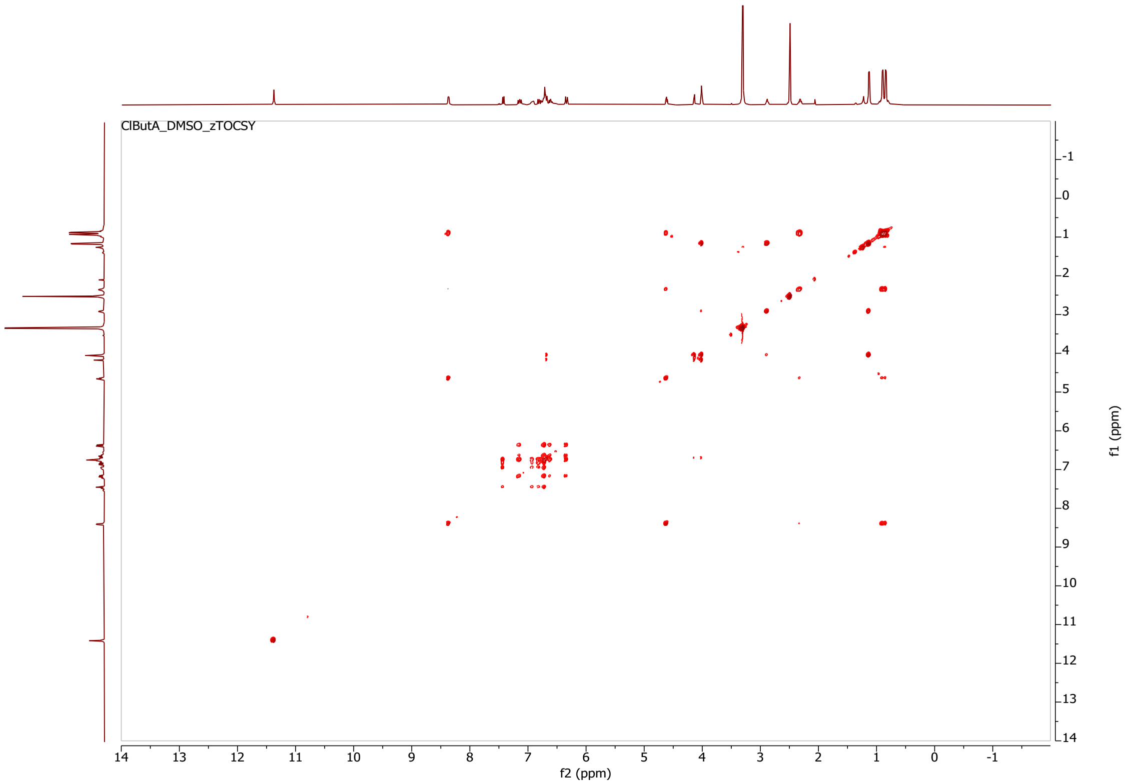

Figure S14. zTOCSY spectrum of **9** in DMSO-d_6 (500 MHz)

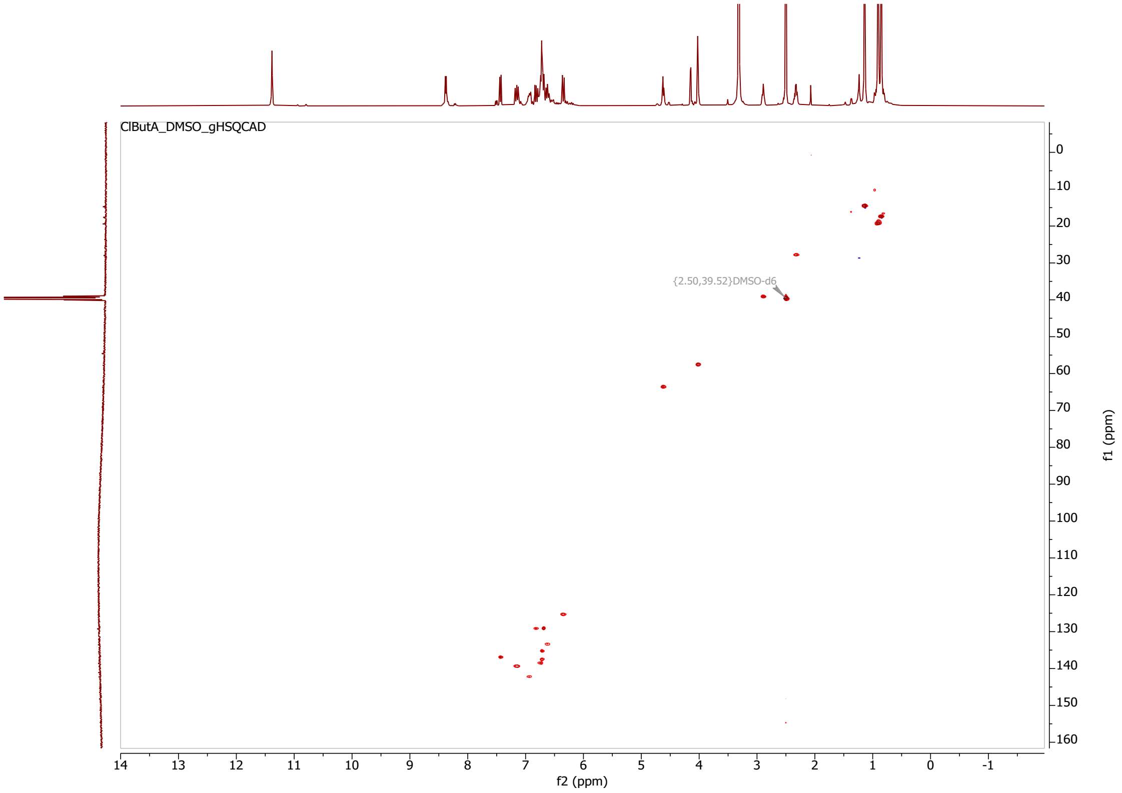

Figure S15. gHSQCAD spectrum of **9** in DMSO-d_6 (500 MHz)

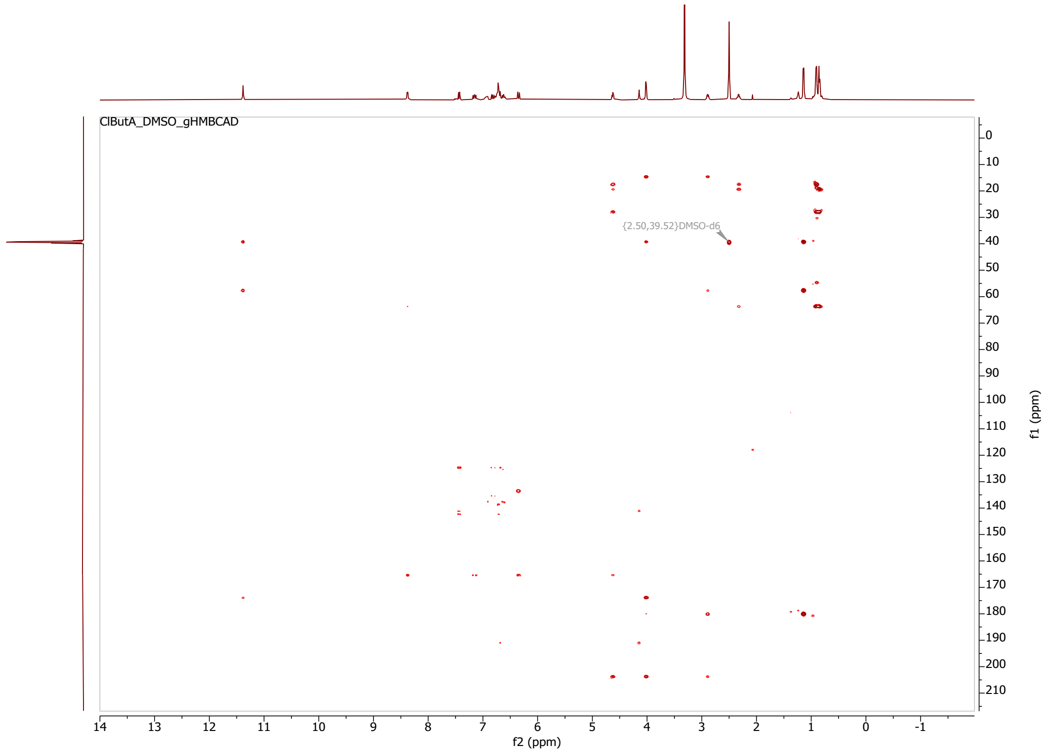

Figure S16. gHMBCAD spectrum of **9** in DMSO-d_6 (500 MHz)

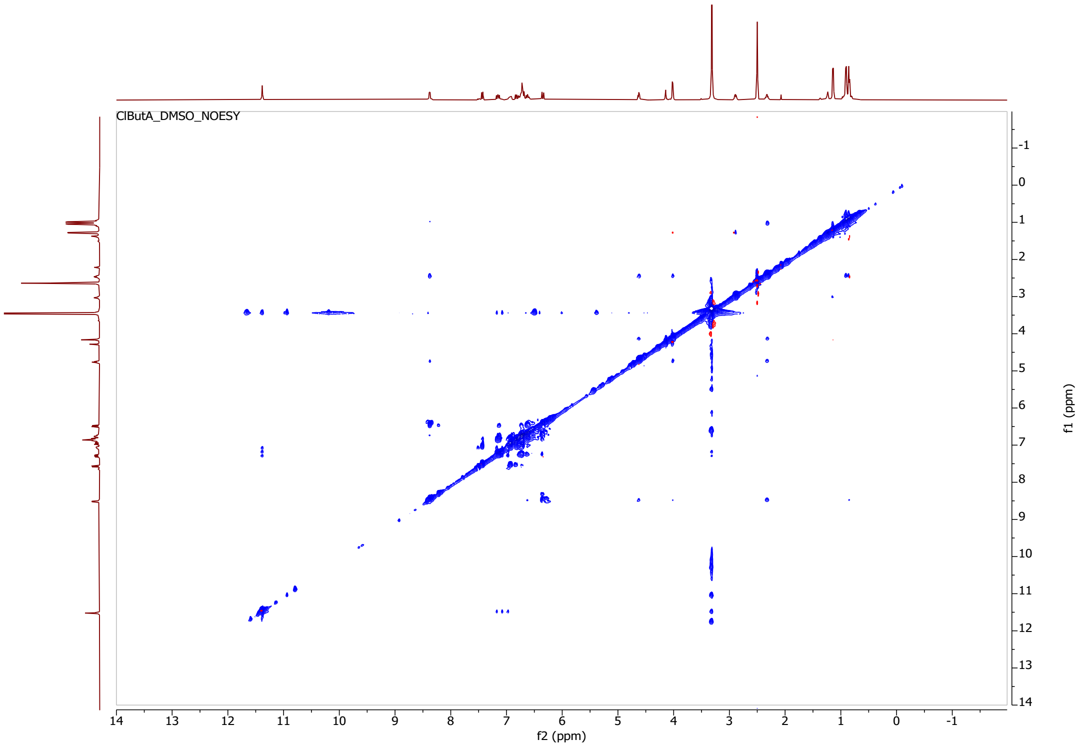

Figure S17. NOESY NMR spectrum of **9** in DMSO-d_6 (500 MHz)

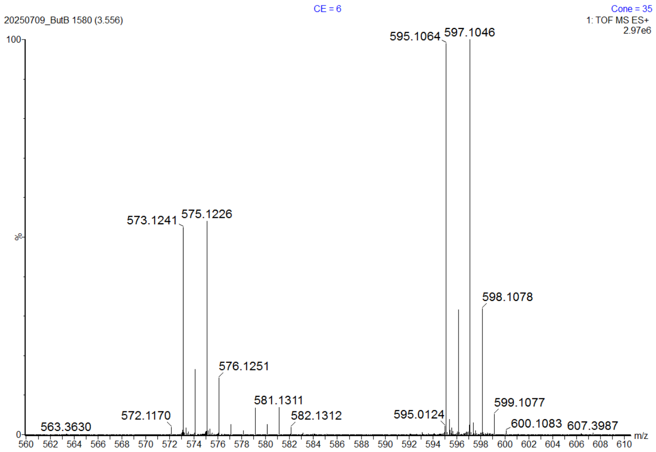

Figure S18. HRESIMS spectrum of **10**

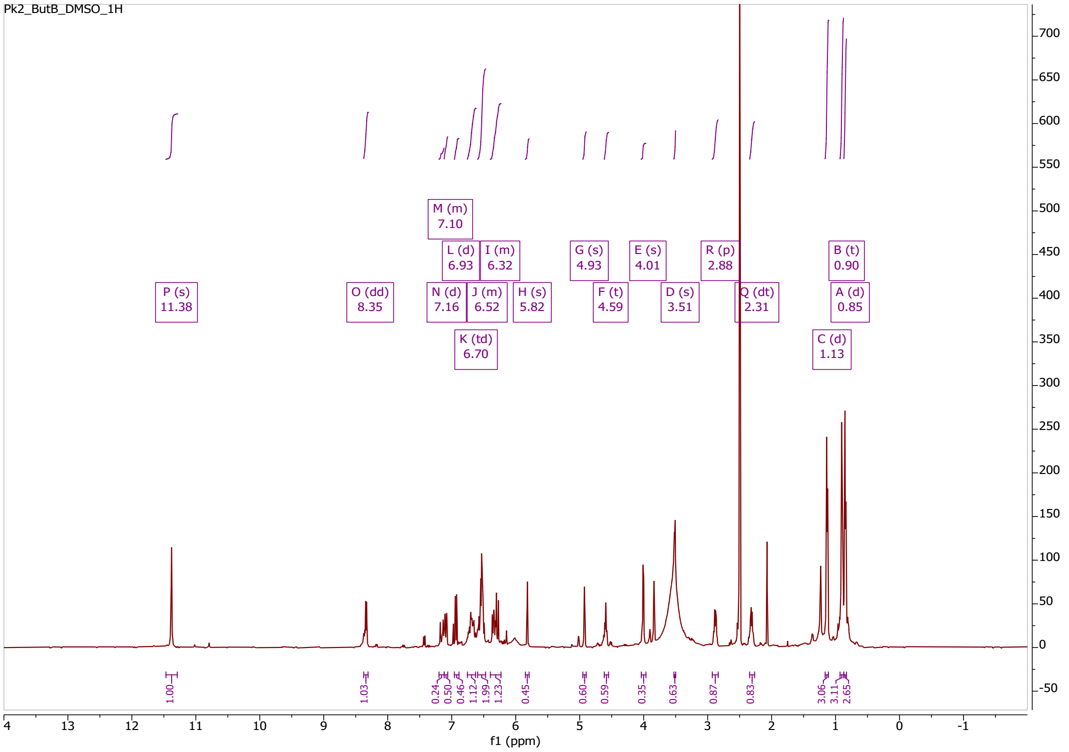

Figure S19. ^{1}H NMR spectrum of **10** in DMSO-d_6 (500 MHz)

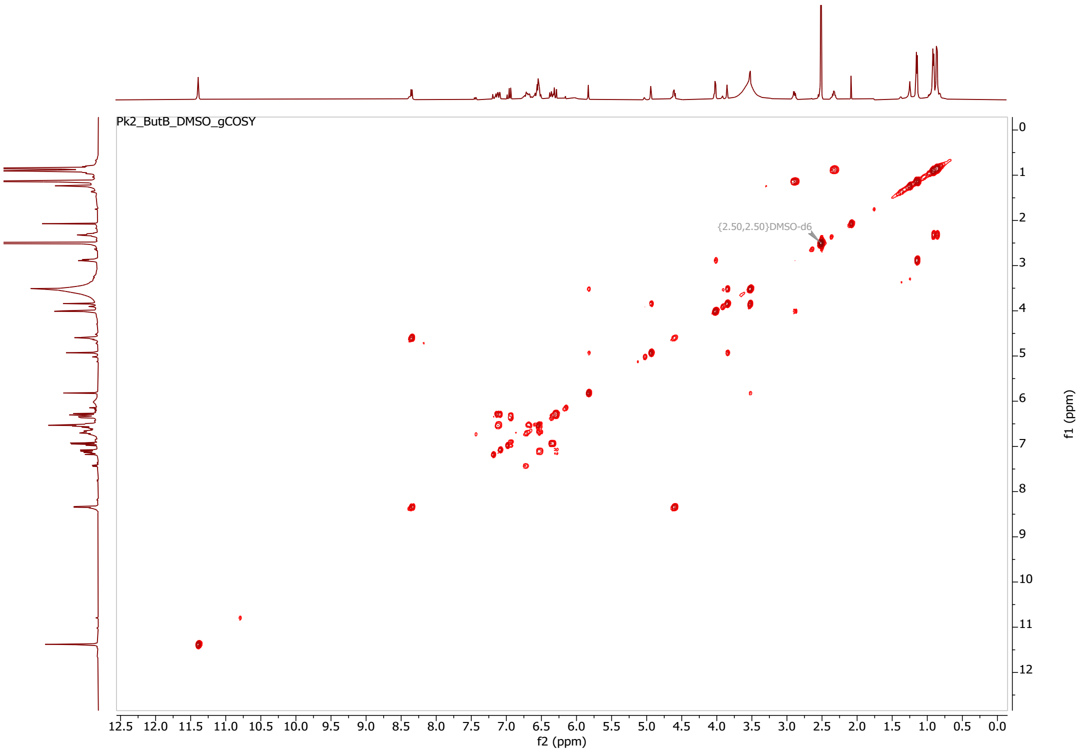

Figure S20. gCOSY spectrum of **10** in DMSO-d_6 (500 MHz)

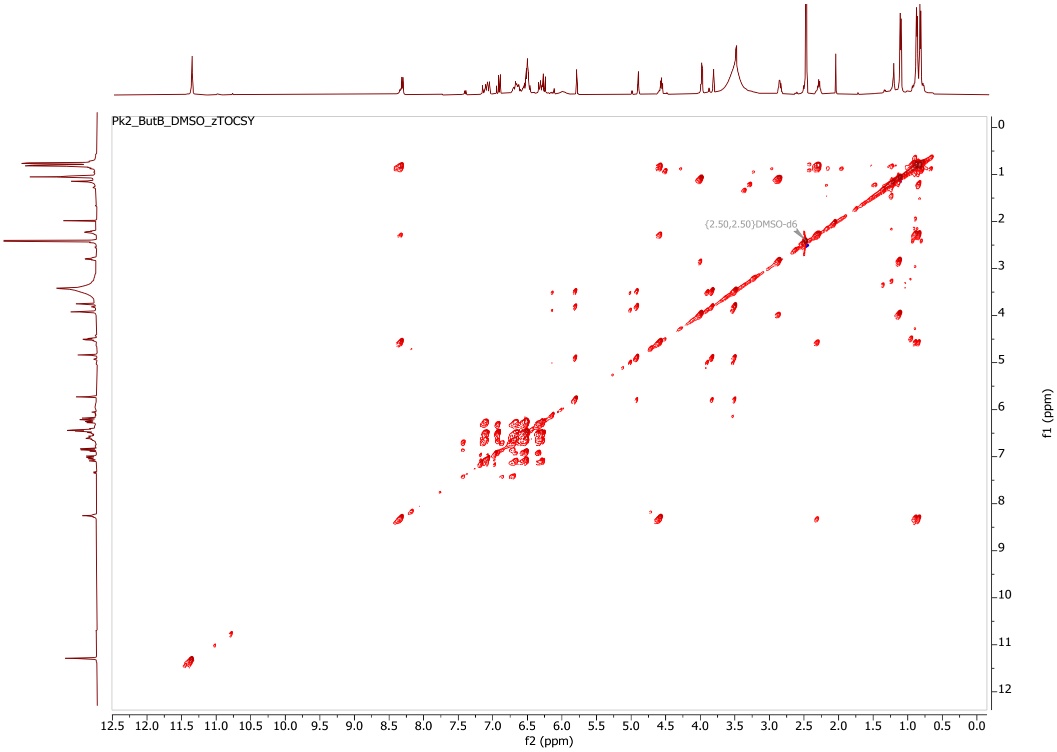

Figure S21. zTOCSY spectrum of **10** in DMSO-d_6 (500 MHz)

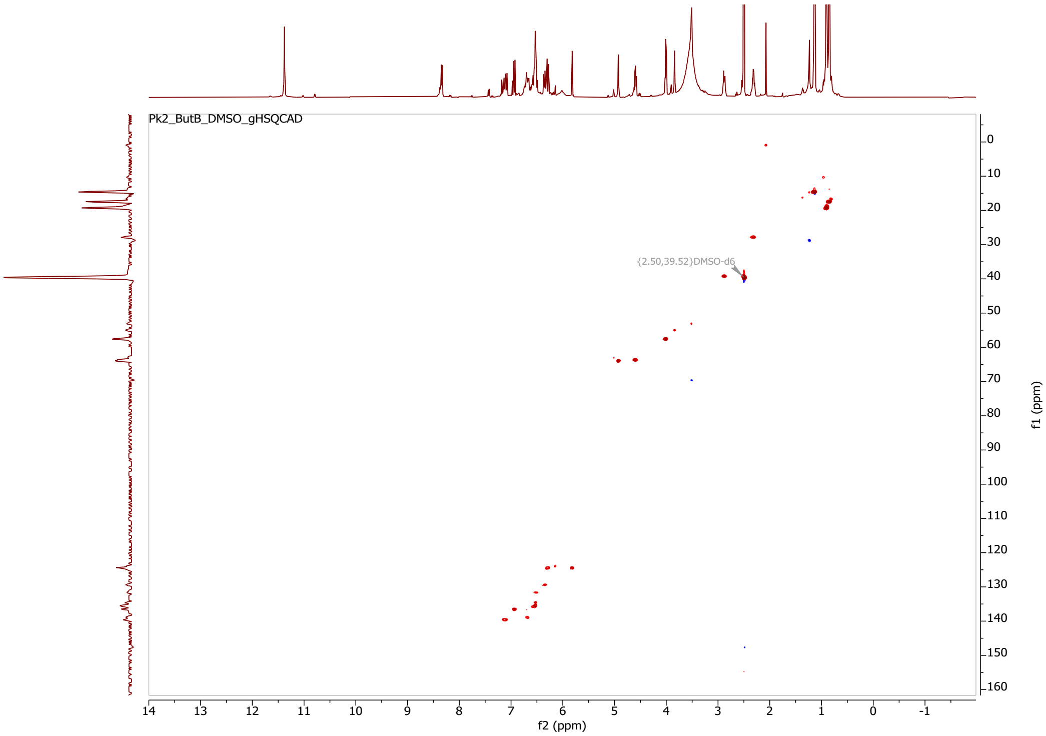

Figure S22. gHSQCAD spectrum of **10** in DMSO-d_6 (500 MHz)

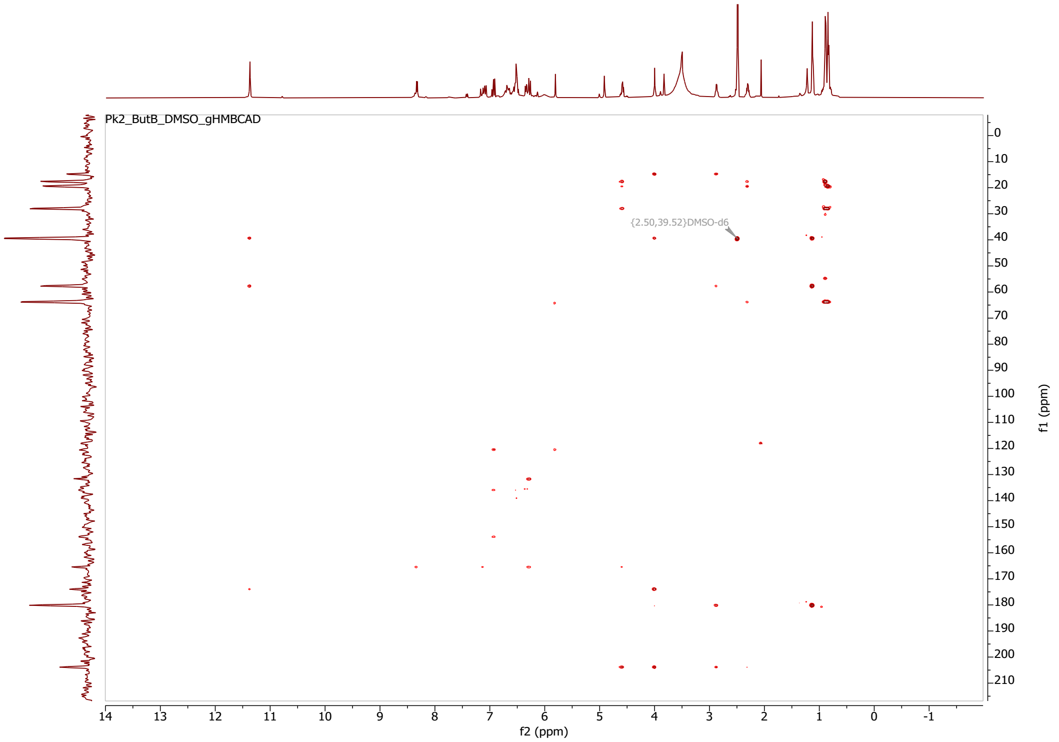

Figure S23. gHMBCAD spectrum of **10** in DMSO-d_6 (500 MHz)

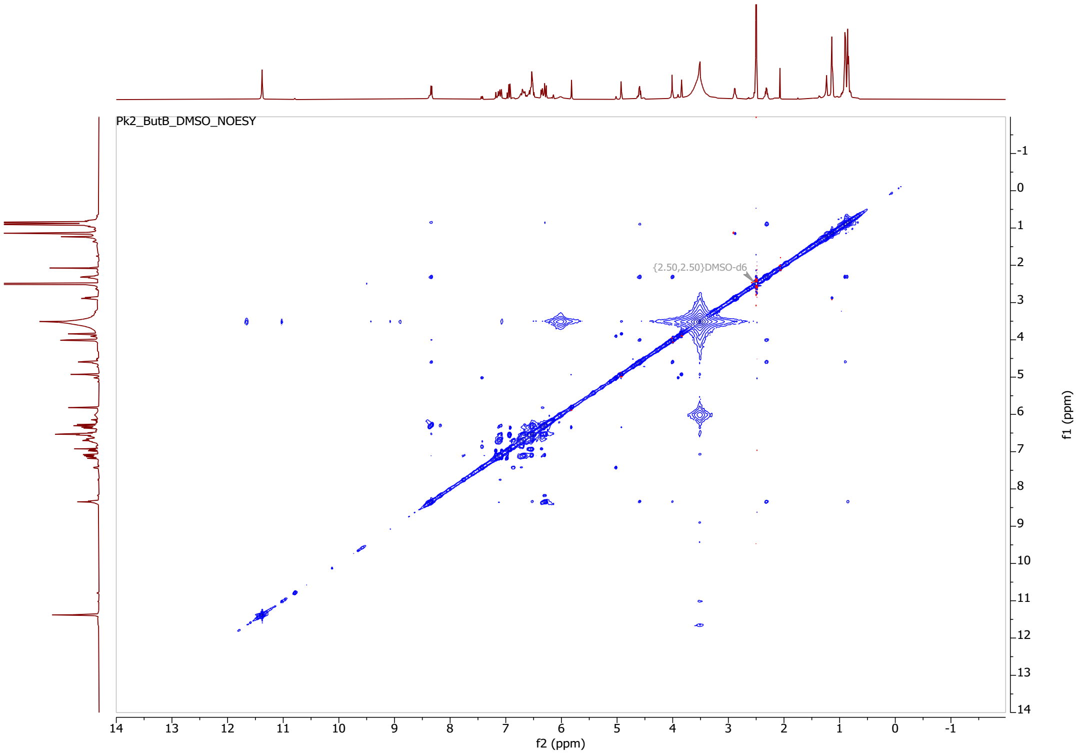

Figure S24. NOESY NMR spectrum of **10** in DMSO-d_6 (500 MHz)

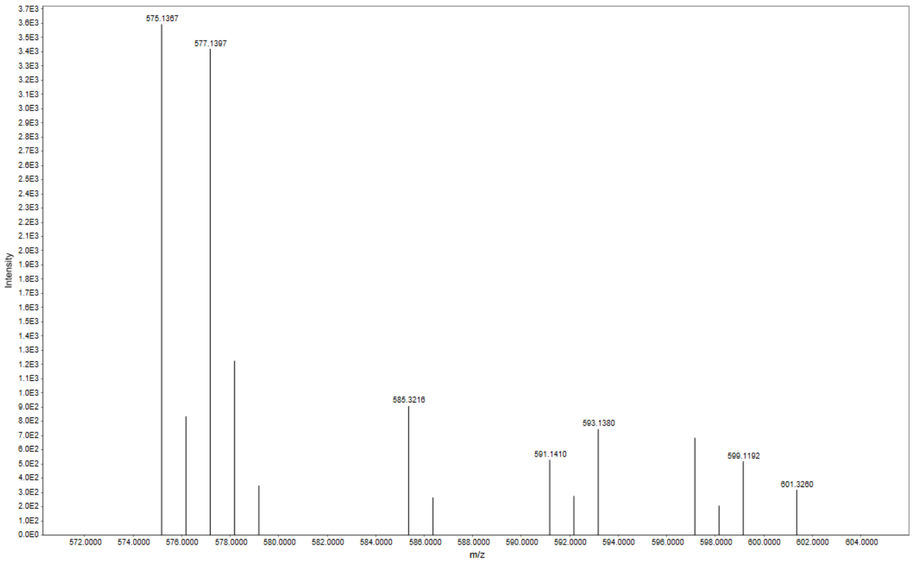

Figure S25. HRESIMS spectrum of **11**

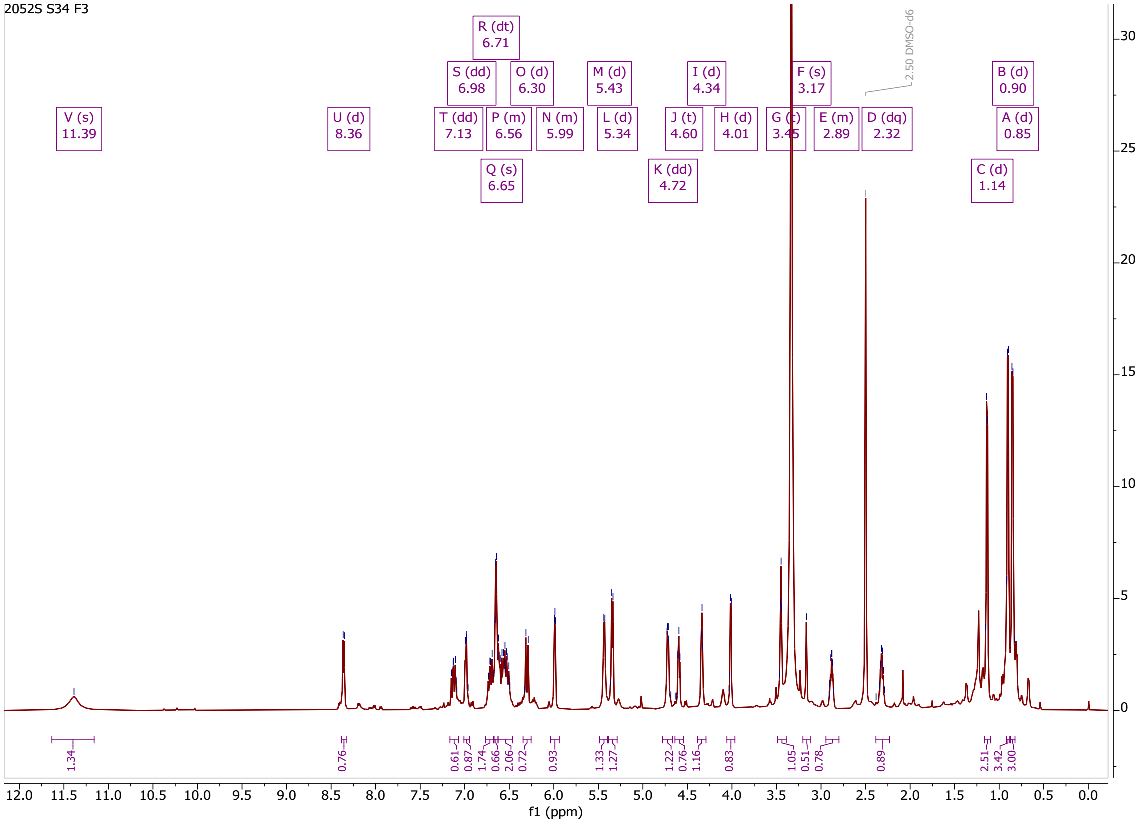

Figure S26. ^{1}H NMR spectrum of **11** in DMSO-d_6 (500 MHz)

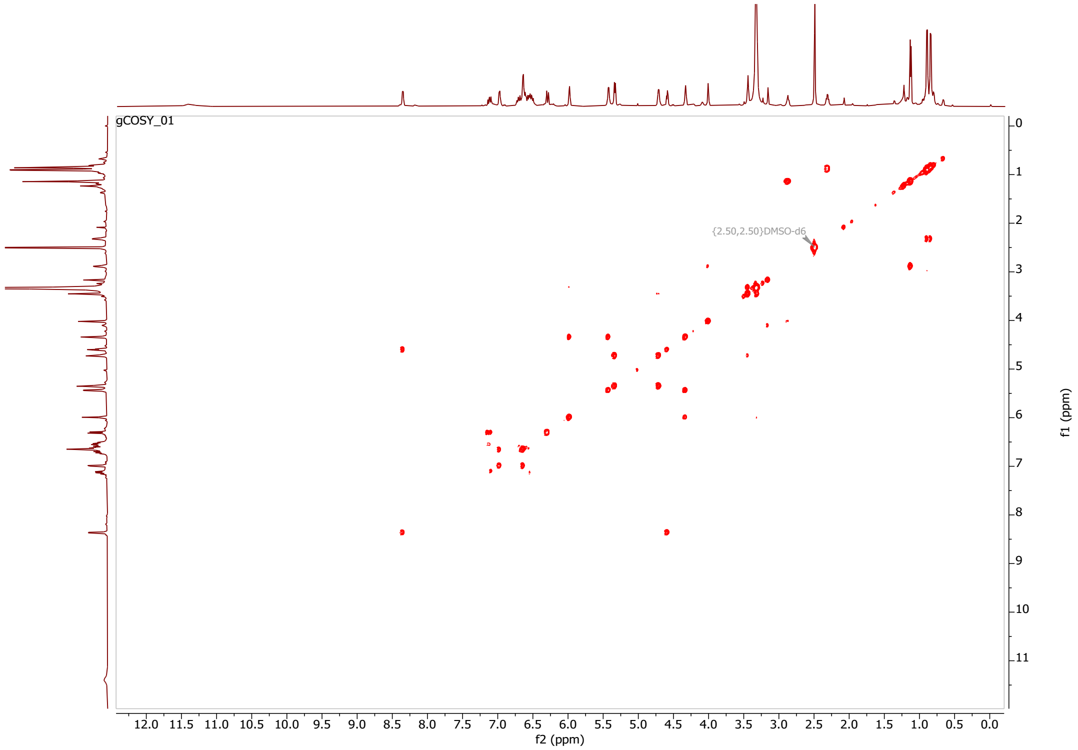

Figure S27. gCOSY spectrum of **11** in DMSO-d_6 (500 MHz)

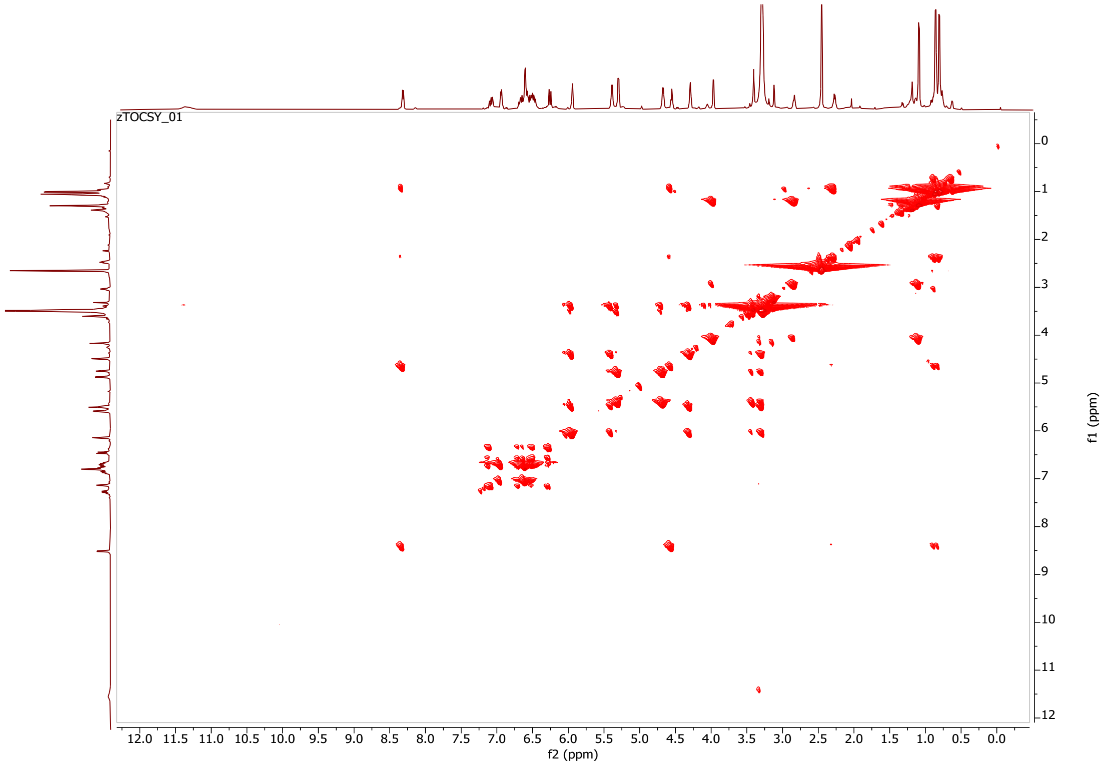

Figure S28. zTOCSY spectrum of **11** in DMSO-d_6 (500 MHz)

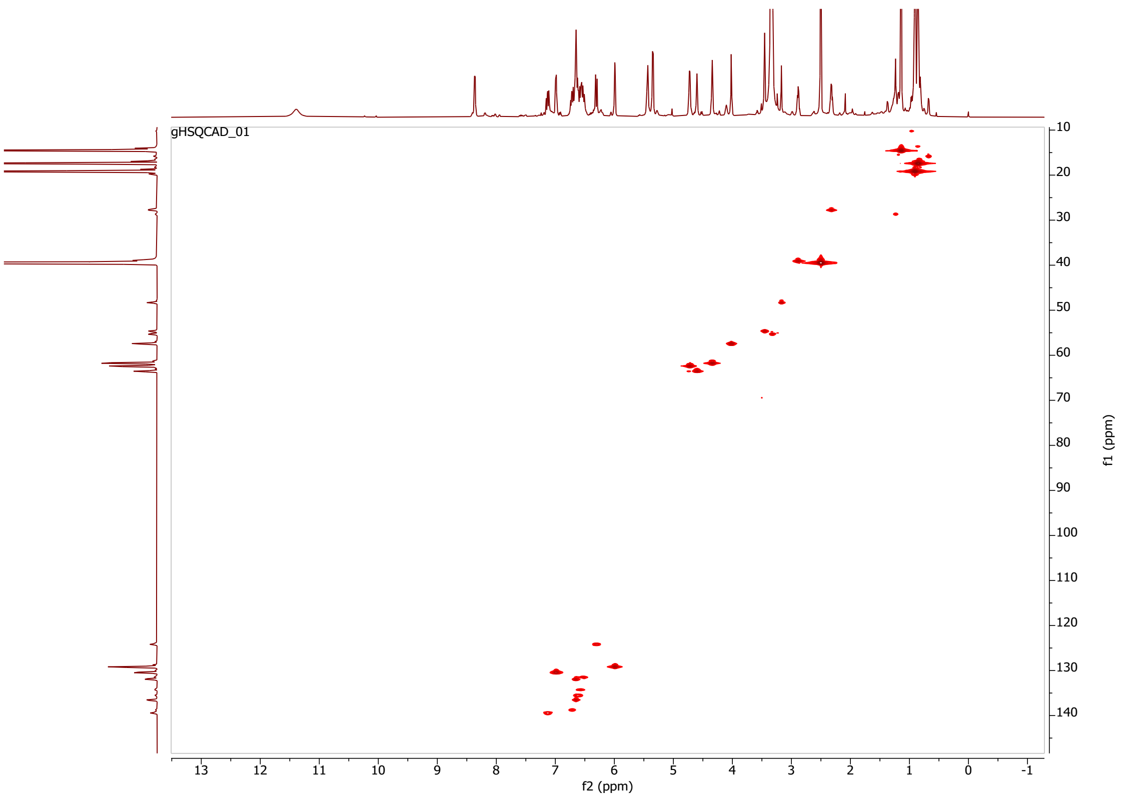

Figure S29. gHSQCAD spectrum of **11** in DMSO-d_6 (500 MHz)

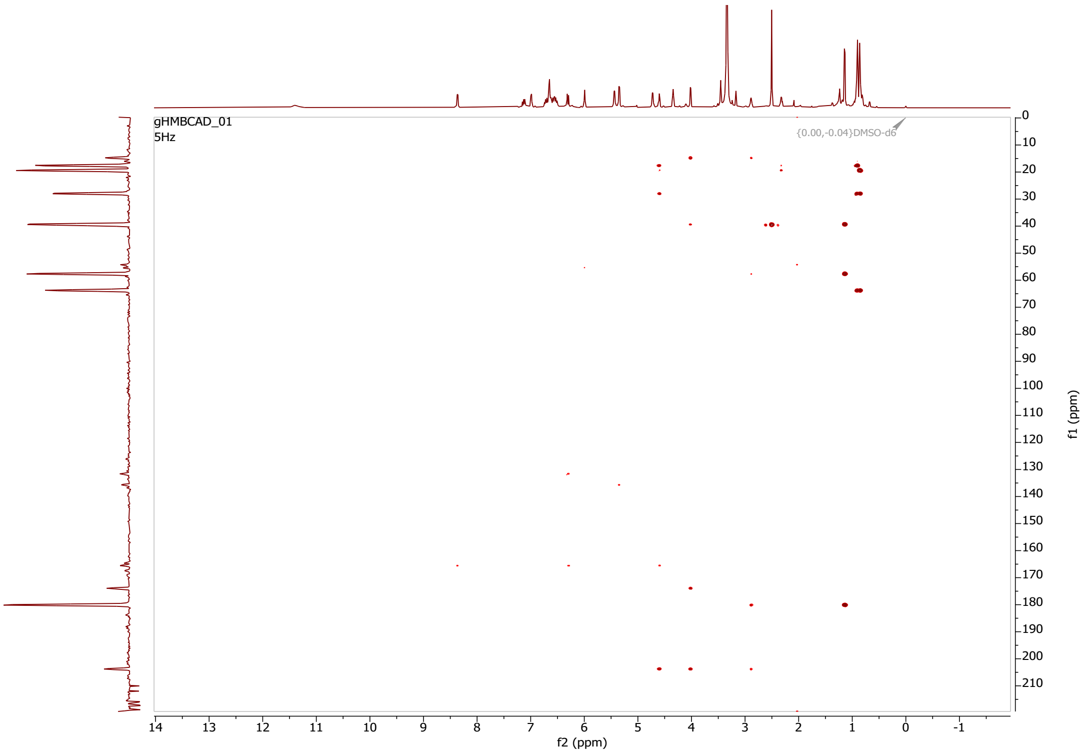

Figure S30. gHMBCAD (5 Hz coupling) spectrum of **11** in DMSO-d_6 (500 MHz)

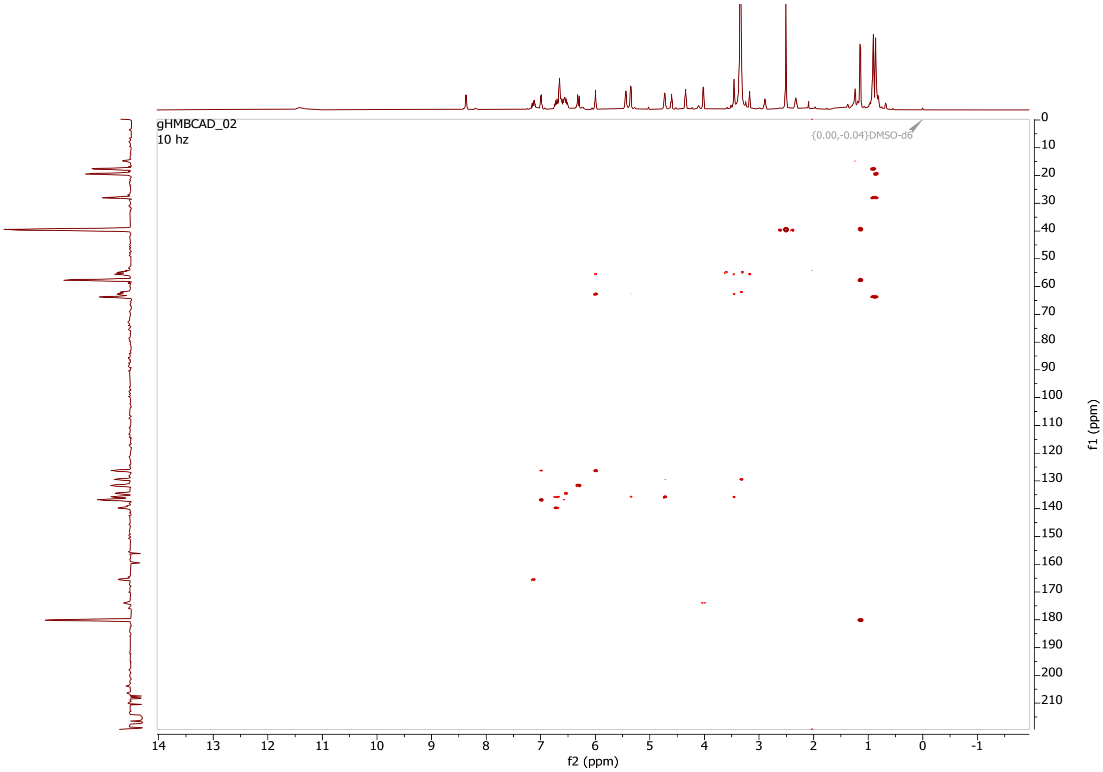

Figure S31. gHMBCAD (10 Hs coupling) spectrum of **11** in DMSO-d_6 (500 MHz)

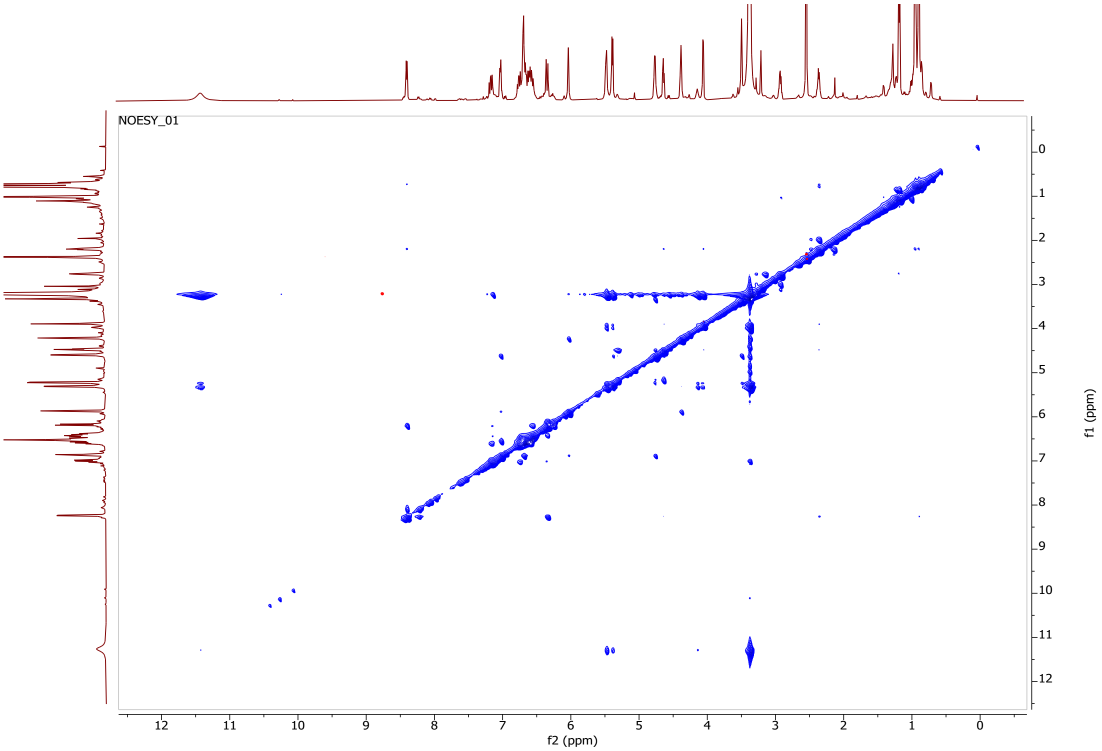

Figure S32. NOESY NMR spectrum of **11** in DMSO-d_6 (500 MHz)

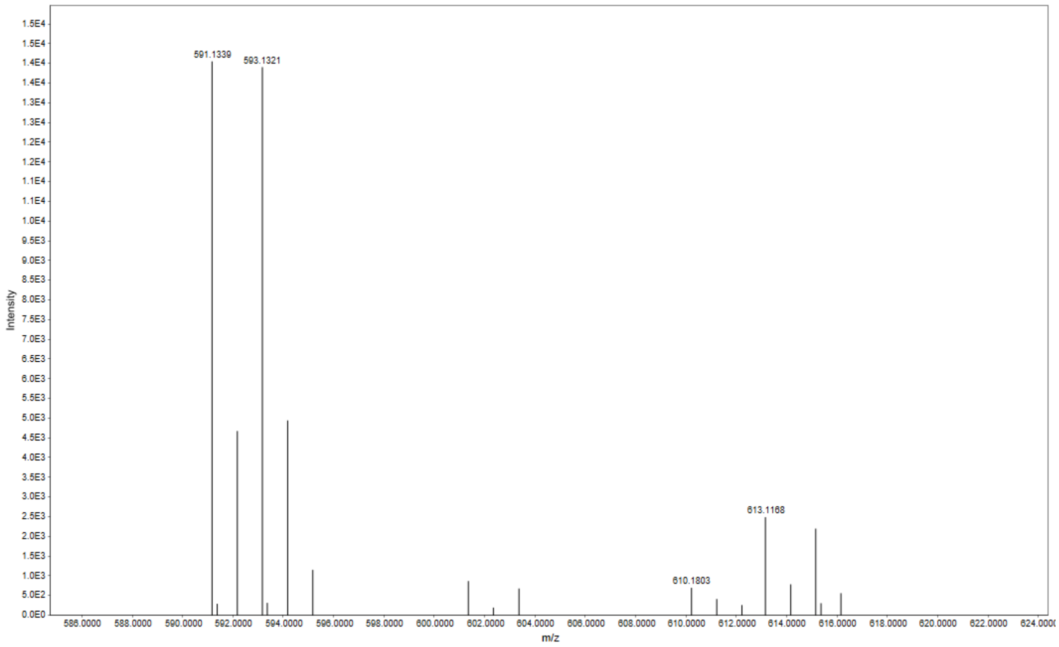

Figure S33. HRESIMS spectrum of **12**

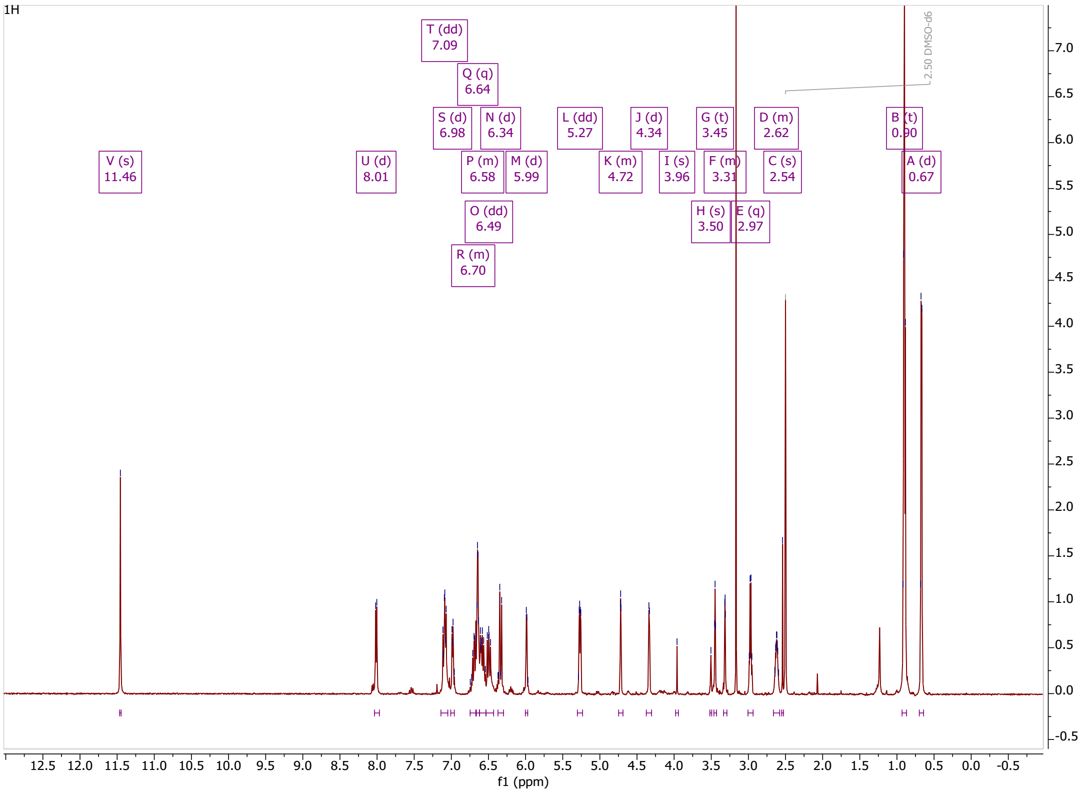

Figure S34. ^{1}H NMR spectrum of **12** in DMSO-d_6 (500 MHz)

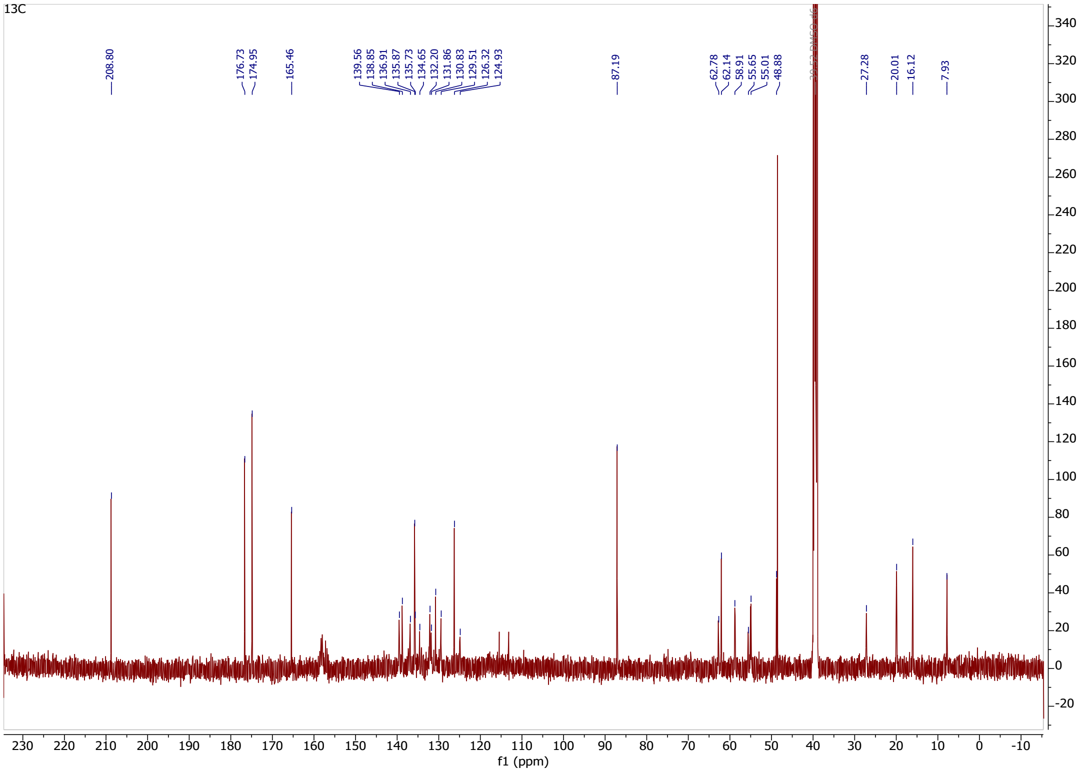

Figure S35. ^{13}C NMR spectrum of **12** in DMSO-*d*6 (125 MHz)

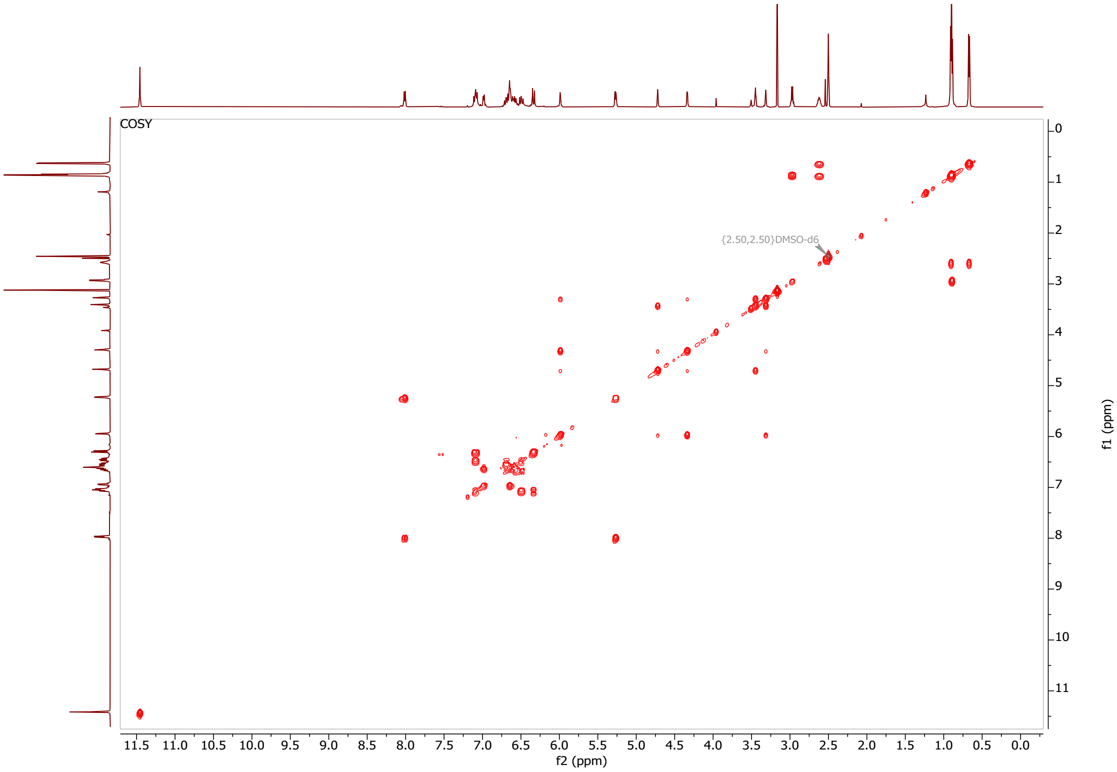

Figure S36. gCOSY spectrum of **12** in DMSO-*d*6 (500 MHz)

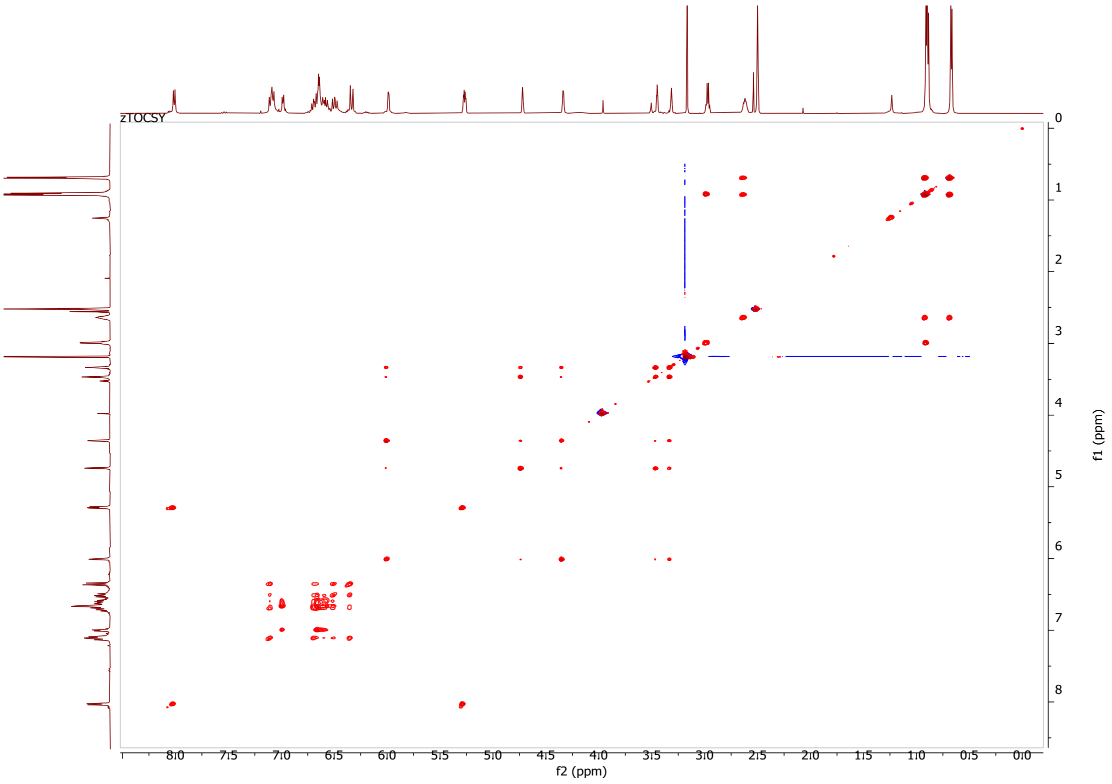

Figure S37. zTOCSY spectrum of **12** in DMSO-d_6 (500 MHz)

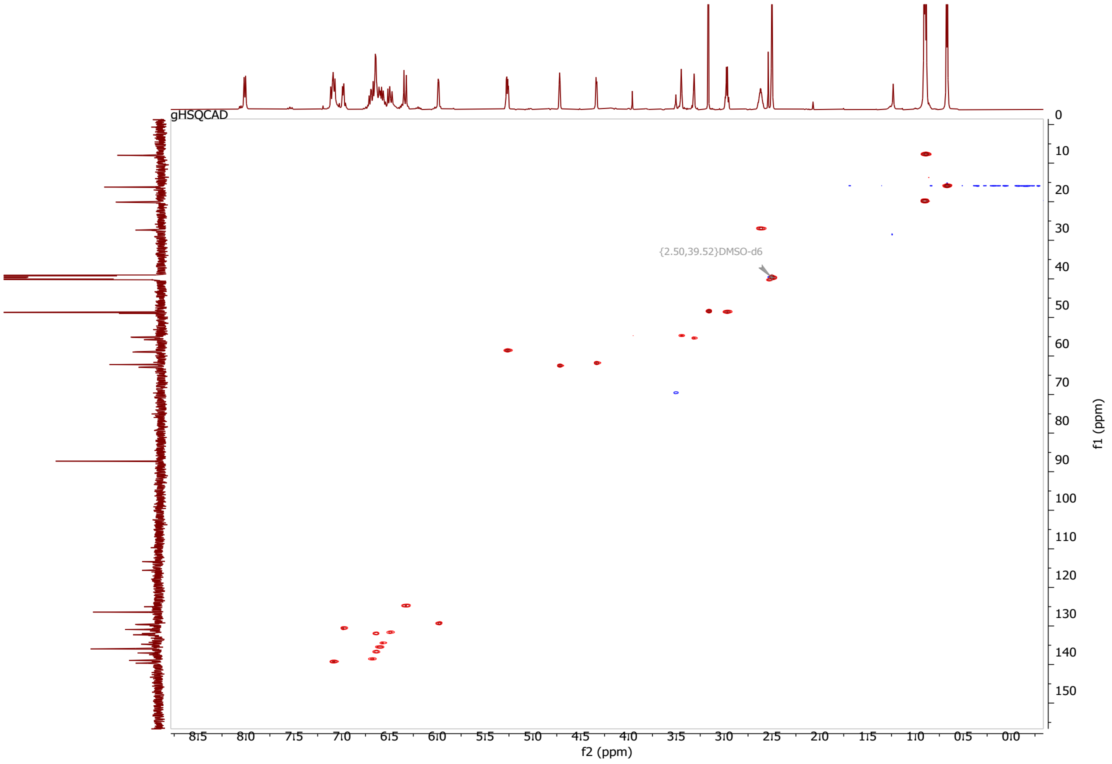

Figure S38. gHSQCAD spectrum of **12** in DMSO-d_6 (500 MHz)

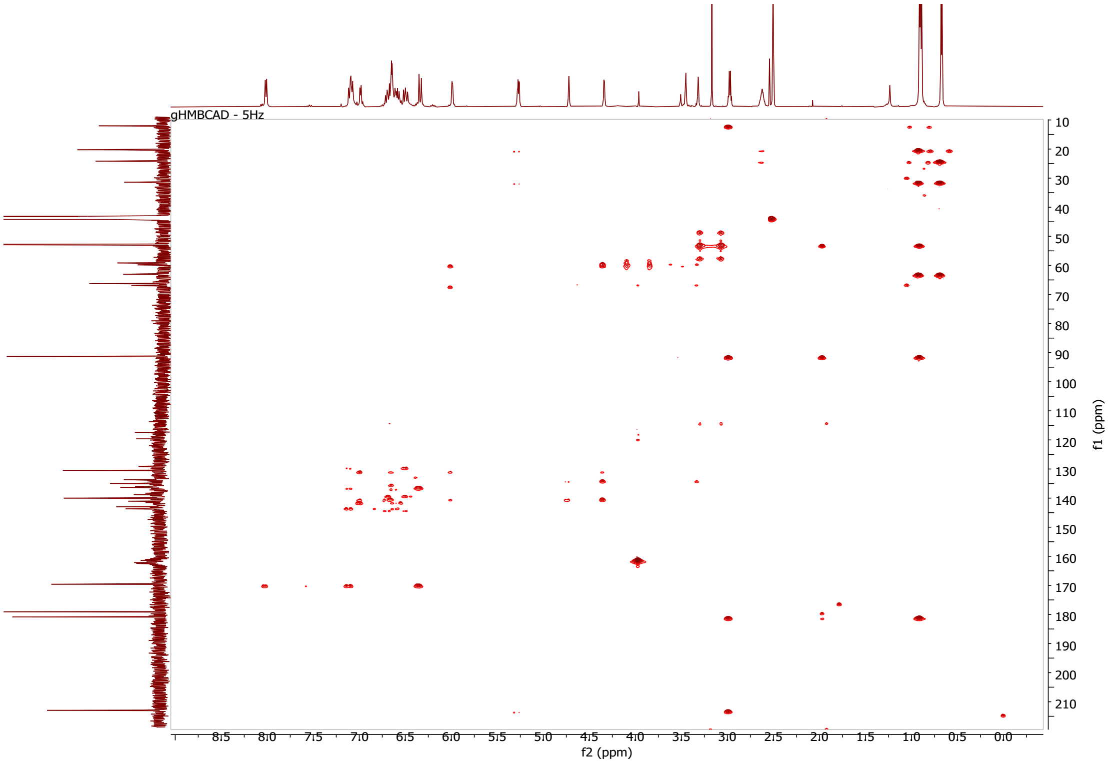

Figure S39. gHMBCAD spectrum of **12** in DMSO-d_6 (500 MHz)

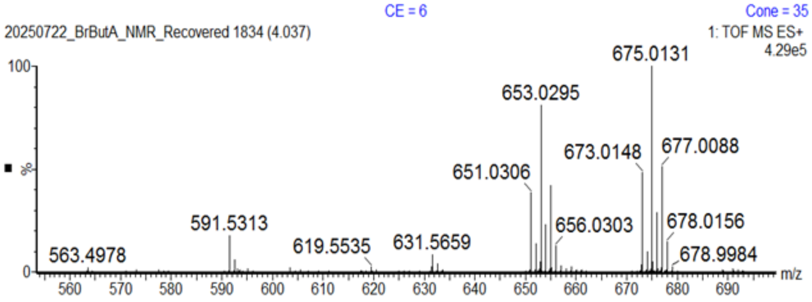

Figure S40. HR-MS spectrum of degradation production found from recovery of NMR sample of **8**.

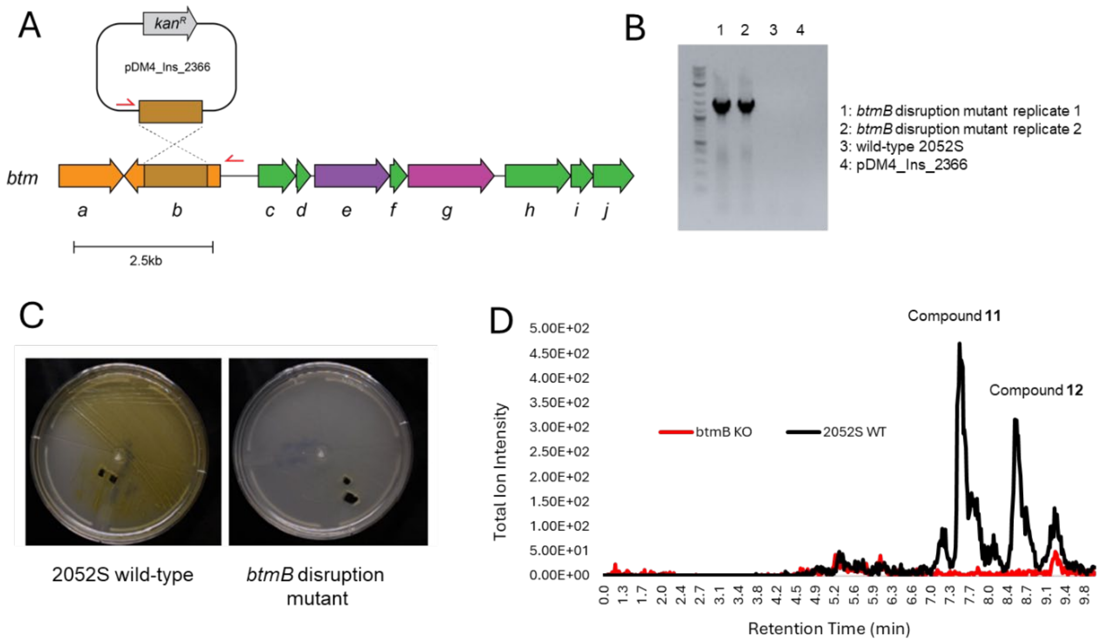

Figure S41. Gene disruption of *btmB* ablates production of butuanimides. A) recombinant engineering approach for disrupting an early biosynthetic enzyme (*btmB*) in the *btm* BGC. B) recombination was confirmed with PCR using one primer in the 2052S chromosome and one in the recombinant plasmid. Bands will only be visible when the plasmid backbone has been integrated into the *btm* BGC. C) *btmB* gene disruption ablates production of pigmented compounds. D) LCMS of 2052S WT (black) and *btmB* disruption shows a loss of production of compounds **11** and **12.** It is noted that these cultures were not grown with resin in the culture, and thus compounds **8**-**10** were not produced.

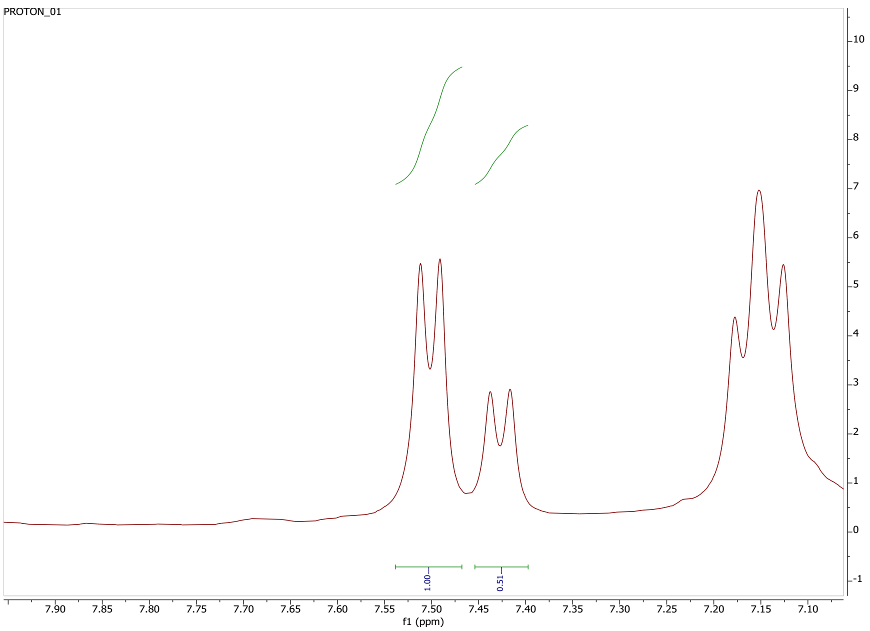

Figure S42. ^{1}H NMR integration of H-22 of mixture of **8** and **9** used in bioassays.

Table S1. ^{13}C and ^{1}H NMR chemical shift comparisons between bromo-butuanimide A (**8**) and andrimid (**1**).

	^{13}C			^{1}H		
	**8**	**1**	**delta**	**8**	**1**	**delta**
1-NH				11.38	11.35	-0.03
2	174.0	173.6	-0.4			
3	57.8	57.8	0	4.02	3.92	-0.1
4	39.4	38.9	-0.5	2.89	2.91	0.02
5	180.2	179.9	-0.3			
6	14.8	14.5	-0.3	1.14	1.07	-0.07
7	203.8	203.3	-0.5			
8	63.8	63	-0.8	4.63	4.63	0
9	28.0	28.1	0.1	2.33	2.29	-0.04
10, 11	17.6	17.2	-0.4	0.85	0.74	-0.11
10, 11	19.5	19.3	-0.2	0.91	0.8	-0.11

Table S2. NMR assignments for compound **8**

**Bromo-butuanimide A (8)**			
**Unit**	**Position**	**δc, Type**	**δ_H, mult (*J* in hz)**
Methyl succinimide	1-NH		11.38, S
	2	174.0, C=O	-
	3	57.8, CH	4.02, d*
	4	39.4, CH	2.89, dt (7.3, 5.3)
	5	180.2, C=O	-
	6	14.8, CH3	1.14, d (7.3)
Valine	7	203.8, C=O	-
	8	63.8, CH	4.63, dd (7.8, 6.1)
	9	28.0, CH	2.33, m (6.6)
	10, 11	17.6, CH3	0.85, d (6.8)
	10, 11	19.5, CH3	0.91, d (6.6)
	12-NH		8.38, d (7.9)
Polyene chain	13	165.5, C=O	-
	14	125.5, CH	6.35, d (15.1)
	15	139.5, CH	7.15, dd (15.0, 11.4)
	16	133.6, CH	6.63, dd (14.3, 11.2)
	17	131-138, CH	6.63-6.78
	18	131-138, CH	6.63-6.78
	19	131-138, CH	6.63-6.78
	20	142.5, CH	6.96, m
	21	131-138, CH	6.72*
	22	139.9, CH	7.51, d (10.6)
	23	117.6, C	-
Epoxyquinone	24	142.8, C	-
	25	131.3, CH	6.67, d (2.4)
	26	190.7, C=O	-
	27	54.6, CH	4.01, m
	28	54.6, CH	4.15, d (4.2)
	29	191.5, C=O	-

*Overlapping signals, assigned by COSY/TOCSY/HMBC/HSQC

Table S3. NMR assignments for compound **9**

**Chloro-butuanimide A (9)**			
**Unit**	**Position**	**δ_C**	**δ_H, mult (*J* in hz)**
Methyl succinimide	1-NH		11.38, S
	2	174, C=O	-
	3	57.8, CH	4.02, d
	4	39.4, CH	2.89, dt (7.3, 5.3)
	5	180.2, C=O	-
	6	14.8, CH3	1.14, d (7.3)
Valine	7	203.8, C=O	-
	8	63.9, CH	4.63, dd (7.8, 6.1)
	9	28.1, CH	2.33, m
	10, 11	17.7, CH3	0.85, d (6.8)
	10, 11	19.5, CH3	0.91, d (6.6)
	12-NH		8.38, d (7.9)
Polyene chain	13	165.5, C=O	-
	14	125.5, CH	6.35, d (15.1)
	15	139.6, CH	7.15, dd (15.0, 11.4)
	16	133.6, CH	6.62, dd (14.3, 11.2)
	17	131-138, CH	6.63-6.78
	18	131-138, CH	6.63-6.78
	19	131-138, CH	6.63-6.78
	20	142.3, CH	6.93, m
	21	129.3, CH	6.81, dd (14.6, 10.8)
	22	137.1, CH	7.43, d (10.6)
	23	124.7, C	-
Epoxyquinone	24	141.1, C	-
	25	129.3, CH	6.69, d (2.4)
	26	190.8, C=O	-
	27	54.7, CH	4.01, m
	28	54.6, CH	4.15, d (4.2)
	29	191.4, C=O	-

Table S4. NMR assignments for compound **10**

**Butuanimide B (10)**			
**Unit**	**Position**	**δc, Type**	**δ_H, mult (*J* in hz)**
Methyl succinimide	1-NH	-	11.38, S
	2	174, C=O	-
	3	57.8, CH	4.01, d (5.7)
	4	39.4, CH	2.88, p (7.2)
	5	180.2, C=O	-
	6	14.8, CH3	1.13, d (7.2)
Valine	7	203.8, C=O	-
	8	63.7, CH	4.59, t (7.1)
	9	28.1, CH	2.33, m
	10, 11	17.5, CH3	0.85, d (6.8)
	10, 11	19.3, CH3	0.90, d (6.6)
	12-NH	-	8.35, d (7.9)
Polyene chain	13	165.5, C=O	
	14	124.5, CH	6.29, d (15.1)
	15	139.6, CH	7.11, dd (15.0, 11.4)
	16	131-138, CH	6.45-6.60
	17	131-138, CH	6.45-6.60
	18	131-138, CH	6.45-6.60
	19	131-138, CH	6.45-6.60
	20	135.9, CH	6.57
	21	129.4, CH	6.34, m*
	22	136.5, CH	6.93, d (11.1)
	23	120.5, C	-
Epoxyquinol	24	154, C	-
	25	124.5, CH	5.82, s
	26	194.0, C=O	-
	27	53.1, CH	3.51, m
	28	55.1, CH	3.84, t (3.6)
	29	64	4.93, s

*Overlapping signals, assigned by COSY/TOCSY/HMBC/HSQC

Table S5. NMR assignments for compound **11**

**Butuanimide C (11)**			
**Unit**	**Position**	**δc, Type**	**δ_H, mult (*J* in hz)**
Methyl succinimide	1-NH	-	11.39, S (br)
	2	173.9, C=O	-
	3	57.4, CH	4.02, d (5.1)
	4	39.1, CH	2.89, m
	5	180.1, C=O	-
	6	14.5, CH3	1.14, d (7.3)
Valine	7	203.6, C=O	-
	8	63.6, CH	4.60, t (7.0)
	9	27.8, CH	2.32, m
	10, 11	17.4, CH3	0.86, d (6.7)
	10, 11	19.2, CH3	0.91, d (6.6)
	12-NH	-	8.36, d (7.8)
Polyene chain	13	165.5, C=O	-
	14	124.2, CH	6.31, d (15.0)
	15	139.5, CH	7.13, dd (15.0, 11.4)
	16	131.5, CH	6.53, dd (14.7, 11.5)
	17	131-138, CH	6.63-6.78
	18	131-138, CH	6.63-6.78
	19	131-138, CH	6.63-6.78
	20	131-138, CH	6.63-6.78
	21	131.9, CH	6.65*
	22	130.5, CH	6.98, d (10.4)
	23	126.4, C	-
Epoxyhydroquinone	24	135.8, C	-
	25	129.2, CH	5.99, s (br)
	26	61.8, CH	4.35, t (br, 5.4)
	27	55.3, CH	3.31*
	28	54.7, CH	3.45, t (3.6)
	29	62.4, CH	4.72, dd (6.4, 3.0)

*Overlapping signals, assigned by COSY/TOCSY/HMBC/HSQC

Table S6. NMR assignments for compound **12**

**Butuanimide D (12)**			
**Unit**	**Position**	**δc, Type**	**δ_H, mult (*J* in hz)**
Methyl succinimide	1-NH		11.46, S
	2	174.9, C=O	-
	3	87.2, C	-
	4	48.8, CH	2.97, q (6.9)
	5	176.7, C=O	
	6	7.9, CH3	0.89, d (7.4)
Valine	7	208.8, C=O	-
	8	58.9, CH	5.27, dd (9.2, 3.6)
	9	27.3, CH	2.62, m
	10, 11	16.1, CH3	0.67, d (6.9)
	10, 11	20.0, CH3	0.90, d (7.4)
	12-NH	-	8.01, d (9.2)
Polyene chain	13	165.5, C=O	-
	14	124.9, CH	6.34, d (14.9)
	15	139.6, CH	7.09, dd (15.0, 11.4)
	16	131.9, CH	6.49, dd (14.7, 11.5)
	17	131-138, CH	6.63-6.78
	18	131-138, CH	6.63-6.78
	19	131-138, CH	6.63-6.78
	20	131-138, CH	6.63-6.78
	21	132.2CH	6.66*
	22	130.8, CH	6.98, d (8.7)
	23	126.3, C	-
Epoxyhydroquinone	24	135.9, C	-
	25	129.5, CH	5.99, d (4.4)
	26	62.1, CH	4.34, d (4.7)
	27	55.7, CH	3.31, m
	28	55.0, CH	3.45, t (3.5)
	29	62.8, CH	4.72, m

*Overlapping signals, assigned by COSY/TOCSY/HMBC/HSQC

Table S7. Comparison of ^{1}H and ^{13}C NMR chemical shifts of **12** and **3**.

		Butuanimide D (**12**)		Moiramide C (**3**)	
Unit	Position	^{1}H	^{13}C	^{1}H	^{13}C
Methyl succinimide	1-NH	11.46, S		11.42, bs	
	2	-	174.9	-	174.8
	3	-	87.2	-	86.8
	4	2.97, q (6.9)	48.8	2.89, q (7.1)	48.6
	5		176.7	-	176.6
	6	0.89, d (7.4)	7.9	0.71. d (7.1)	7.7
Valine	7	-	208.8	-	208.6
	8	5.27, dd (9.2, 3.6)	58.9	5.18, dd (9.4, 3.4)	58
	9	2.62, m	27.3	2.50, m	27.4
	10, 11	0.67, d (6.9)	16.1	0.61, d (6.8)	15.9
	10, 11	0.90, d (7.4)	20	0.81, d (6.8)	19.7
	12-NH	8.01, d (9.2)	-	7.83. d (9.4)	-

Table S8. Annotations of gene in *btm* biosynthetic gene cluster, including identity comparison to nearest *adm* gene homologs.

**Query**	**Adm Analog (% ID)**	**Top BLAST hit**	**Organism**	**Top Hit Accession**	**%ID**
BtmA		FAD-dependent monooxygenase	*Aliikangiella coralliicola*	ref|WP_142893842.1|	47
BtmB		NAD(P)/FAD-dependent oxidoreductase	Gammaproteobacteria bacterium	gb|MEE8261752.1|	46
BtmC		beta-ketoacyl synthase chain length factor	*Empedobacter haloabium*	gb|WUR14328.1|	35
BtmD		acyl carrier protein	*Massilia* sp. YMA4	ref|WP_170289092.1|	38
BtmE		AMP-binding protein	*Pseudoduganella plicata*	ref|WP_134387101.1|	37
BtmF		MAG: hypothetical protein AUJ57_03150	Zetaproteobacteria bacterium CG1_02_53_45	gb|OIO74023.1|	36
BtmG	AdmH (32%)	HAL/PAL/TAL family ammonia-lyase	*Pseudoduganella plicata*	ref|WP_134387103.1|	65
BtmH		beta-ketoacyl-ACP synthase	*Massilia* sp. YMA4	ref|WP_112938427.1|	49
BtmI		hypothetical protein E7V67_004315	*Empedobacter haloabium*	gb|WUR14334.1|	37
BtmJ	AdmC (35%)	3-oxoacyl-ACP reductase FabG	*Pseudoduganella plicata*	ref|WP_134387106.1|	57
BtmK		MATE family efflux transporter	*Agarilytica* sp.	gb|MFL0802923.1|	46
BtmL	AdmK (38%)	non-ribosomal peptide synthetase	*Serratia quinivorans*	ref|WP_368459915.1|	41
BtmM	AdmS (37%)	transglutaminase-like domain-containing protein	*Serratia quinivorans*	ref|WP_368459917.1|	53
BtmN	AdmL (56%)	ester cyclase	*Serratia plymuthica*	ref|WP_062867881.1|	59
BtmO	AdmM (43%)	beta-ketoacyl synthase N-terminal-like domain-containing protein	*Serratia quinivorans*	ref|WP_368459919.1|	45
BtmP	AdmN (50%)	Dabb family protein	*Vibrio anguillarum*	ref|WP_194662280.1|	55
BtmQ	AdmO (40%)	beta-ketoacyl synthase N-terminal-like domain-containing protein	*Serratia quinivorans*	ref|WP_368459922.1|	42
BtmR	AdmP (39%)	AMP-binding protein	*Serratia quinivorans*	ref|WP_368459924.1|	40
BtmS		cupin domain-containing protein	*Massilia* sp. YMA4	ref|WP_112938415.1|	39
BtmT		acyl-CoA dehydrogenase family protein	*Empedobacter haloabium*	gb|WUR14346.1|	59
BtmU		efflux RND transporter permease subunit	Gammaproteobacteria bacterium	gb|MDH5232693.1|	54
BtmV		outer membrane lipoprotein-sorting protein	Gammaproteobacteria bacterium	gb|MDH5232694.1|	55
BtmW		hypothetical protein	Gammaproteobacteria bacterium	gb|MDH5232695.1|	34
BtmX		sulfite exporter TauE/SafE family protein	*Teredinibacter waterburyi*	ref|WP_245792232.1|	45
BtmY		4'-phosphopantetheinyl transferase family protein	*Pseudoalteromonas obscura*	ref|WP_284138789.1|	48
BtmZ		NAD(P)-dependent dehydrogenase (short-subunit alcohol dehydrogenase family)	Alteromonadaceae bacterium 2753L.S.0a.02	gb|TVZ41538.1|	70
BtmAA		DNA-binding transcriptional LysR family regulator	Alteromonadaceae bacterium 2753L.S.0a.02	gb|TVZ41539.1|	75

Table S9. Strains and plasmids used in this study

Strains	Description	Reference
E. coli DH5α	*fhuA2::IS2 Δ(mmuP-mhpD)169 ΔphoA8 glnX44 ϕ80d[ΔlacZ58(M15)] rfbD1 gyrA96 luxS11 recA1 endA1 rphWT thiE1 hsdR17*	New England Biolabs
E. coli S17-1 λpir	Donor strain. Tpr Smr *recA thi pro hsd*(r-m+) RP4-2-Tc::Mu::Km Tn7 λpir	[3]
**Plasmid**	**Description**	**Reference**
pGEM-T Easy	Cloning vector containing the f1 origin of replication	Promega
pDM4-Km	Suicide vector used to construct gene mutants of Gram-negative bacteria. SacB gene, R6K origin, Kanamycin^r	[4]
pDM4_Ins_2366	pDM4-Km containing a 1.5kb insert amplified from btmB	This study

Table S10. Cloning and diagnostic primers used in this study

Primer	Sequence (5' – 3')[a]	Description
btmB_F_SpeI	ACTAGTCGACATCATCATTATCGGTGGC	For amplifying 1.5kb pcr fragment within btmB to insert into pDM4
btmB_R_1.5kb_SmaI	CCCGGGGACTGTGTTTGCCATCAGCGAC	
btmB_disruption_pDM4_F	TTTGCGTAACGGCAAAAGCAC	For confirming successful disruption mutant amplifying. Forward primer is from pDM4_Ins_2366 and reverse primer is 50bp upstream of btmB
btmB_disruption_2366_R	AATTATCGCCGGGTGGAGCAG	

[a]Restriction enzymes sites are underlined.

References

(1) Waterbury, J. B.; Calloway, C. B.; Turner, R. D. A Cellulolytic Nitrogen-Fixing Bacterium Cultured from the Gland of Deshayes in Shipworms (Bivalvia: Teredinidae). *Science* **1983**, *221* (4618), 1401–1403. https://doi.org/10.1126/science.221.4618.1401.

(2) Gilchrist, C. L. M.; Chooi, Y.-H. Clinker & Clustermap.Js: Automatic Generation of Gene Cluster Comparison Figures. *Bioinformatics* **2021**, *37* (16), 2473–2475. https://doi.org/10.1093/bioinformatics/btab007.

(3) Simon, R.; Priefer, U.; Pühler, A. A Broad Host Range Mobilization System for In Vivo Genetic Engineering: Transposon Mutagenesis in Gram Negative Bacteria. *Nat Biotechnol* **1983**, *1* (9), 784–791. https://doi.org/10.1038/nbt1183-784.

(4) Milton, D. L.; O'Toole, R.; Horstedt, P.; Wolf-Watz, H. Flagellin A Is Essential for the Virulence of Vibrio Anguillarum. *Journal of Bacteriology* **1996**, *178* (5), 1310–1319. https://doi.org/10.1128/jb.178.5.1310-1319.1996.
